# Differences in the plasma phospholipid profile of patients infected with tick-borne encephalitis virus and co-infected with bacteria

**DOI:** 10.1038/s41598-022-13765-2

**Published:** 2022-06-09

**Authors:** Monika Groth, Wojciech Łuczaj, Justyna Dunaj-Małyszko, Elżbieta Skrzydlewska, Anna Moniuszko-Malinowska

**Affiliations:** 1 Department of Infectious Diseases and Neuroinfections, Medical University of Białystok, Żurawia 14, 15-540 Białystok, Poland; 2 Department of Analytical Chemistry, Medical University of Bialystok, Mickiewicza 2d, 15-222 Bialystok, Poland

**Keywords:** Biochemistry, Infection

## Abstract

Tick-borne encephalitis (TBE) is an infectious viral disease, the pathogenesis of which is still not fully understood. Additionally, TBE can be complicated by co-infections with various bacteria that are also transmitted by ticks, which can affect the proper diagnosis and treatment. Therefore, the aim of the study was to evaluate changes in the plasma phospholipid (PL) and ceramide (CER) profile of patients with TBE and patients with bacterial co-infection (*B. burgdorferi* or *A. phagocytophilum*) in relation to healthy subjects. For this purpose, a high-resolution LC-QTOF-MS/MS platform as well as univariate and multivariate statistics were used. The results of this study showed that the levels of phosphatidylcholines (PC) and lysophosphatidylcholines (LPC) species were increased in the plasma of patients with TBE and patients with TBE co-infected with bacteria. On the other hand, observed differences in the content of phosphoethanolamines (PE) and sphingomyelins (SM) make it possible to distinguish TBE patients from patients with co-infections. The opposite direction of changes was also observed in the CER content. This study showed significant modifications to the metabolic pathways of linoleic (LA) and arachidonic acid (AA), as confirmed by the quantitative analysis of these fatty acids. The obtained results allow to distinguish the pathomechanism of TBE from TBE with bacterial co-infection, and consequently may improve the diagnostic process and enable more efficient pharmacotherapy against both pathogens.

## Introduction

TBE is a vector-borne disease endemic in Europe caused by TBE virus (TBEV) which belongs to *Flaviviridae* family. TBEV is transmitted by *Ixodes ricinus* ticks, which are common in Europe. The number of TBE cases in Europe and Asia varies depending on country and ranges from a few cases up to 10,000–12,000 cases per year^1^. TBE may take clinical course of meningitis, meningoencephalitis or meningoencephalomyelitis^2^. Meningitis is usually mild and patients recover after 2–3 weeks of symptomatic treatment. In patients with meningoencephalitis and meningoencephalomyelitis the course of the disease is more severe. Unfortunately, the pathogenesis of TBE is still not fully clarified. However, it is well known that the infection caused by TBEV promotes immune deregulation associated with pro-inflammatory reactions, which shift cellular redox homeostasis to oxidative processes^3^. Such situation usually leads to oxidative stress, which is accompanied by an increase in oxidative modifications of main cellular components, including lipids, and in particular polyunsaturated fatty acids (PUFAs). PLs are the main reservoirs of PUFAs, as precursors for lipid mediators and signaling molecules^4^. PUFAs are believed to be involved in the development of viral infections, including COVID-19, and involvement of AA in the passivation of viruses has been also suggested^5^. Our previous study has shown enhanced PL peroxidation in the course of TBE^6^. Consequently, elevated levels of PUFAs oxidative fragmentation products, such as small molecule aldehydes, including malondialdehyde (MDA), 4-hydroxy-2-nonenal (4-HNE) and 4-hydroxy-2-hexenal (4-HHE), and PUFA oxidative cyclization products such as isoprostanes and neuroprostans were observed in plasma, cerebrospinal fluid and urine of patients with tick-borne encephalitis.

In order to comprehensively assess the changes in the structure and functions of PLs, omics studies have been used for several years, which have been proven especially useful in the development of new diagnostic and therapeutic tools in the case of diseases with ambiguous clinical picture^7,8^. To date, only one metabolomics study has been published indicating PL species that may be involved in metabolic pathways altered in TBE^3^. Despite the data presented in the literature, the pathogenesis of TBE, as well as its relationship with lipid metabolism is still not fully understood. The situation is further complicated by the fact that tick is a vector of various pathogens, not only TBEV, but also many bacteria, including *Borrelia burgdorferi* sensu lato, *A. phagocytophilum* and protozoa *Babesia* spp. which may lead to co-infection^9^. This may result in changes in the pathomechanism of the disease and different spectrum of clinical symptoms making the diagnostic process and pharmacotherapy more challenging. Since non-specific symptoms are observed in TBE as well as Lyme disease (LD) caused by *Borrelia burgdorferi* and human granulocytic anaplasmosis (HGA) caused by *Anaplasma phagocytophilum*, co-infection with these diseases should be considered in the differential diagnosis, especially in patients residing or with travel history to areas endemic for tick-borne diseases^10,11^.

Considering the above, a better understanding of metabolic changes, particularly those related to PLs in the course of TBE, especially in the case of co-infection with other pathogens (in particular bacterial infection), is of key importance for the correct diagnosis and effective pharmacotherapy. Therefore, the aim of the study was to assess lipidomic changes accompanying both the development of TBE and bacterial co-infections with *B. burgdorferi* or *A. phagocytophilum*. We used LC-QTOF-MS/MS based lipidomics to analyze differences and similarities in the plasma PL and CER profiles of patients with TBE and TBE patients with bacterial co-infections, as well as healthy subjects.

## Materials and methods

### Samples collection

Blood samples were obtained from 16 patients with tick-borne encephalitis (5 female and 11 men), mean age: 40 (age range: 23–58) and 6 patients with TBE co-infection with other tick-borne pathogens including *Borrelia burgdorferi* (LD) or *A. phagocytophilum* infection (4 female and 2 men, mean age: 45 (age range: 22–63) treated in the Department of Infectious Diseases and Neuroinfections, Medical University of Bialystok, Poland. TBE was diagnosed according to European Academy of Neurology (EAN) guidelines [EUR-Lex 2021], based on clinical symptoms, positive serology and lymphocytic pleocytosis in the cerebrospinal fluid (CSF). Co-infection was diagnosed if one patient was infected with at least two different pathogens. LD was defined on the basis of clinical presentation of erythema migrans or fulfilled criteria for neuroborreliosis^12,13^. The control group comprised of 8 healthy donors (2 female and 6 men, mean age: 37 (28–50). HGA was diagnosed according to the case definition by CDC when all 3 criteria were fulfilled (https://wwwn.cdc.gov/nndss/conditions/ehrlichiosis-and-anaplasmosis/case-definition): 1. Clinical presentation; 2. Exposure to a tick bite. 3. Laboratory Criteria for Diagnosis: Detection of *A. phagocytophilum* DNA by polymerase chain reaction (PCR) assay.

The study was conducted in accordance with the Declaration of Helsinki, and the protocol for the collection of all blood samples was approved by the Local Bioethics Committee in Medical University of Bialystok (Poland), No. R-I-002/169/2018. Written informed consent was obtained from all participants.

The blood was taken twice: at admission and after treatment. The mean time between these two examinations in TBE patients was 26 ± 12 days, min 7 days, max 43 days, while the mean time between these two examinations in co-infected patients was 33 ± 4 days, min 28 days, max 39 days.

Control group consisted of 13 healthy blood donors from Regional Centre for Transfusion Medicine, Bialystok, Poland.

Blood samples were collected into ethylenediaminetetraacetic acid (EDTA) tubes and next were centrifuged at 3000×*g* (4 °C) for 5 min to obtain the plasma. Aliquots (0.5 mL) of the plasma samples were transferred to Eppendorf tubes and Butylhydroxytoluene (BHT), as an antioxidant was added (at final concentration of 20 μM) to all plasma samples to prevent oxidation. The samples were then stored at –80 °C until analysis.

Demographic and clinical characteristics of patients and control, as well as comparison of their laboratory data are presented in Tables 1 and 2.

**Table 1:** Demographic and clinical characteristics of patients with TBE and co-infections (TBE + LD/HGA) compared to healthy subjects.

	Control	TBE	TBE + LD/HGA
Average age (age range)	37 (28–50)	40 (23–58)	42 (22–63)
Sex (% of participants)	2/8 female (25%) 6/8 male (75%)	5/16 female (33%) 11/16 male (67%)	4/6 female (67%) 2/6 male (33%)
Noticeable tick bite (% of participants)	NA	5/16 (33%)	5/6 (83%)
Time since tick bite (days)	NA	34 ± 24	18 ± 5
Duration of hospitalization (days)	NA	14 ± 2	12 ± 2
Duration of symptoms prior to hospital admission (days)	NA	8 ± 10	4 ± 3
**Clinical form (% of participants)**			
Meningitis	NA	7/16 (44%)	5/6 (83%)
Meningoencephalitis		9/16 (56%)	1/6 (17%)
Meningoencephalomyelitis		0/16 (0%)	0/6 (0%)

*NA* not applicable.

**Table 2:** Comparison of laboratory data of patients with TBE and co-infections (TBE + LD) compared to healthy subjects. Values are means ± SD.

	Normal range	Control Group	TBE	TBE + LD/HGA
			At admission	At admission
**Complete blood count**				
WBC [10^3^/μL]	4.00–10.00	6.35 ± 0.97	10.09 ± 2	7.27 ± 1.36
Neutrophils [%]	40.0–72.0	–	70.8 ± 9.50	60.33 ± 7.07
Lymphocytes [%]	18.0–48.0	–	18.48 ± 8.02	27.37 ± 6.11
Monocytes [%]	2.50–10.00	–	9.55 ± 2.8	10.15 ± 2.61
RBC [10^6^/μL]	4.00–5.50	5 ± 0.31	4.37 ± 0.35	4.28 ± 0.56
HGB [g/dl]	12.00–16.00	14.56 ± 0.94	13.08 ± 1.05	12.67 ± 1.26
PLT [10^3^/μL]	130–350	246.53 ± 50.17	255 ± 51	263 ± 67
CRP [mg/L]	0.00–5.00	–	8.29 ± 14.27	2.69 ± 2.24
Glucose [mg/dL]	70–110	–	99.8 ± 10.7	97 ± 5.66
Creatinine [mg/dL]	0.50–0.90	–	0.90 ± 0.13	0,79 ± 0.08
ALT [U/I]	0–31	–	23 ± 25	18 ± 11
AST [U/I]	0–32	–	16.47 ± 6.35	17 ± 4
**CSF analysis**				
Cytosis [cells/*µl]*	0–5	–	163 ± 99	102 ± 135
Protein [mg/dl]	15–45		75.13 ± 22.30	71.2 ± 65.11

### Extraction of lipids and quantification of PL content

Lipid extracts from plasma samples were obtained using the modified Folch method^14^, by adding 1.5 ml of ice-cold methanol and 3 ml of chloroform to each 200 μl of plasma samples After addition of 1.25 ml H_2_O (miliQ) samples were vortexed and centrifuged at 2500 × g for 10 min at room temperature and the organic (bottom) phase was collected. The PL content of each lipid extract was calculated by the colorimetric phosphorus assay^15^. In short, the method included acidic hydrolysis with perchloric acid followed by incubation with ammonium molybdate in the presence of ascorbic acid. The amount of total PL present in each sample was calculated from the absorbance measured at 800 nm, in a microplate UV–Vis spectrophotometer (Multiskan GO, Thermo Scientific, Hudson, NH, USA). All experimental procedures concerning lipid extraction and PL quantification described in detail in previously published studies^16,17^.

### Determination of free and PL fatty acid level

The plasma level of PL fatty acids was analyzed by GC with a flame ionization detector (FID)^18^. First, the lipids were isolated by Folch extraction using chloroform/methanol mixture (2:1, v/v) in the presence of 0.01% butylated hydroxytoluene (BHT). TLC separated PL fatty acids with the mobile chase heptane—diisoprophyl ether—acetic acid (60:40:3, v/v/v) and transmetylated to fatty acid methyl ester (FAME) with boron trifluoride in methanol reagent under nitrogen atmosphere at 100 °C for 30 min for PLs. Two standards were used, including 1,2-dinonadecanoyl-sn-glycero-3-phosphocholine (PC(19:0/19:0)) (Avanti Polar Lipids, Alabaster, AL, USA) to assess the overall recovery of the extraction method, and methyl nonadecanoate (MeC19:0) (Supelco, North Harrison Road, Bellefonte, PA, USA) as an internal standard (ISTD) for the GC analysis. The FAMES were analyzed by gas chromatography with a flame ionization detector (GC-FID; Clarus 500, PerkinElmer, Shelton, CT, USA) using a capillary column coated with CP-Sil 88 stationary phase (50 m × 0.25 mm × 0.20 μm; Agilent, Palo Alto, CA, USA). The injector and FID detector temperatures were kept at 260 °C. The column temperature was programmed from 150 °C (2 min)–230 °C (10 min) at 4 °C/min. Identification of FAMEs was made by comparison with their retention time with standards.

### PL profiling by hydrophilic interaction liquid chromatography coupled with high-resolution tandem mass spectrometry (HILIC-MS/MS)

PLs were separated by HILIC using a UPLC system (Agilent 1290; Agilent Technologies, Santa Clara, CA, USA) coupled with a QTOF mass spectrometer (Agilent 6540; Agilent Technologies, Santa Clara, CA, USA). Internal standards of PC(14:0/14:0), LPC(19:0), PE(14:0/14:0), PI(16:0/16:0) and PS(14:0/14:0) were used for the quantification and assessment of the ions variations. The mixture composed of solvent A [ACN/MeOH/water 50:25:25 (v/v/v) with 1 mM ammonium acetate] and solvent B [ACN/MeOH 60:40 (v/v) with 1 mM ammonium acetate] was used as mobile phase. The gradient elution was applied started with 0% of A, increased linearly to 100% of A within 20 min and held for 15 min, then returned to 0% of A in 10 min. Twenty-five μg of each PL extract corresponding to a volume of 10 μL was mixed with 90 μL of mobile phase (60% of A and 40% of B). A volume of 10 μL of diluted sample was loaded into the Ascentis® Si column (15 cm × 1 mm, 3 μm, Supelco, Bellefonte, PA, USA) with the mobile phase flow rate of 40 μL per min. The QTOF mass spectrometer operated using a negative-ion mode (electrospray voltage, − 3000 V) with capillary temperature, 250 °C and sheath gas flow of 13 L/min. Data-dependent acquisition mode (DDA) was used for data collecting in the range of *m*/*z* 100–1500 with a fixed collision energy of 35 eV.. All LC–MS/MS analysis was performed in negative ionization mode. Although LPC, PC and SM are sensitive to be detected in positive ion mode, thanks to the addition of ammonium acetate to mobile phase these PLs can be analyzed in the negative mode as adducts with acetate anions [M + CH_3_COO]^-^. However, PI, PE and PS are preferentially analyzed in negative ion mode as deprotonated [M-H]^-^ ions. Furthermore, LC–MS/MS analysis in negative mode used in this study allows identification the fatty acyl residues esterified to the glycerol backbone at sn-1 and sn-2 position seen in the MS spectra after decomposition of molecular ions corresponding to each PL species. MS/MS spectra of some identified PL species are included in supplementary materials. Data acquisition was carried out with the use of Mass Hunter data software (version B0.8.0, Agilent Technologies, Santa Clara, CA, USA). The relative content of each PL ion species was achieved by normalization the area of each peak to the peak area of the corresponding internal standard. The retention times and obtained MS/MS spectra were the basis for PL identification.

### CER profiling by reversed-phase chromatography coupled with high-resolution tandem mass spectrometry RPLC-MS/MS

An Agilent UPLC-ESI-QTOF-MS system (Agilent 1290; Agilent 6540; Agilent Technologies, Santa Clara, CA, USA) was used to characterize the CER profiles. The mobile phase was composed of solvent A (water with 20 mM ammonium formate pH 5) and solvent B (methanol). Initially, 70% of B was held isocratically for 1 min, followed by a linear increase to 100% of B within 75 min, and return to initial conditions in 5 min. The CERs were separated on an RP C18 column (Acquity BEH Shield 2.1 × 100 mm; 1.7 μm; Waters, Milford, MA, USA) with a flow rate of 0.5 mL/min. The QTOF mass spectrometer was operated in positive ion mode (electrospray voltage 3.5 kV) with a capillary temperature of 300 °C and a sheath gas flow rate of 8 L/min. The data was collected in DDA mode. Identification of CER species was based on the presence of the [M + H]^+^ molecular ion, retention time and characteristic fragmentation patterns observed in MS/MS spectra, which was previously described in detail^19^. Cermide internal standards, N-lignoceroyl-d-erythro-sphingosine and N-lignoceroyl-d-erythro-sphinganine (Avanti Polar Lipids, Alabaster, AL, USA) were used for quantification of CERs.

### Data processing

The filtering, peak detection, alignment and integration as well as the assignment of each PL species was carried out by the MZmine 2.30 software for the data obtained^20^.

### Statistical analysis

Univariate and multivariate statistical analyses were performed using Metaboanalyst version 4.0 online tool (https://www.metaboanalyst.ca/)^21^. The data obtained by MS/MS analysis were autoscaled before Principal component analysis (PCA). Univariate statistical analysis was carried out using the ANOVA test with Tukey’s post hoc test with P < 0.05 considered statistically significant. The heatmaps were created using "Euclidean" as the clustering distance and "Ward" as the clustering algorithm.

The Metaboanalyst was also applied for the pathway analysis identified highly enriched metabolic pathway of differential lipids via the Kyoto Encyclopedia of Genes and Genomes (KEGG) database.

## Results

In this study HILIC-LC–MS/MS platform and the RP–HPLC–MS/MS technique were applied to evaluate the changes in plasma PL and CER profiles of TBE patients, TBE patients with co-infection and healthy volunteers. PL species belonging to seven different classes were identified in the analyzed samples. These were PC, PE, phosphatidylinositols (PI), phosphatidylserine (PS), lyso-phosphatidylethanolamine (LPE), lyso- LPC, and SM. The lists of most abundant PL species which were identified and quantified are presented in Supplementary Table S3 (supplementary materials).

### Comparison of the plasma PL profile of healthy subjects (control) and TBE patients (TBE) and TBE patients with co-infections (TBE + LD/HGA)

**Figure 1:**
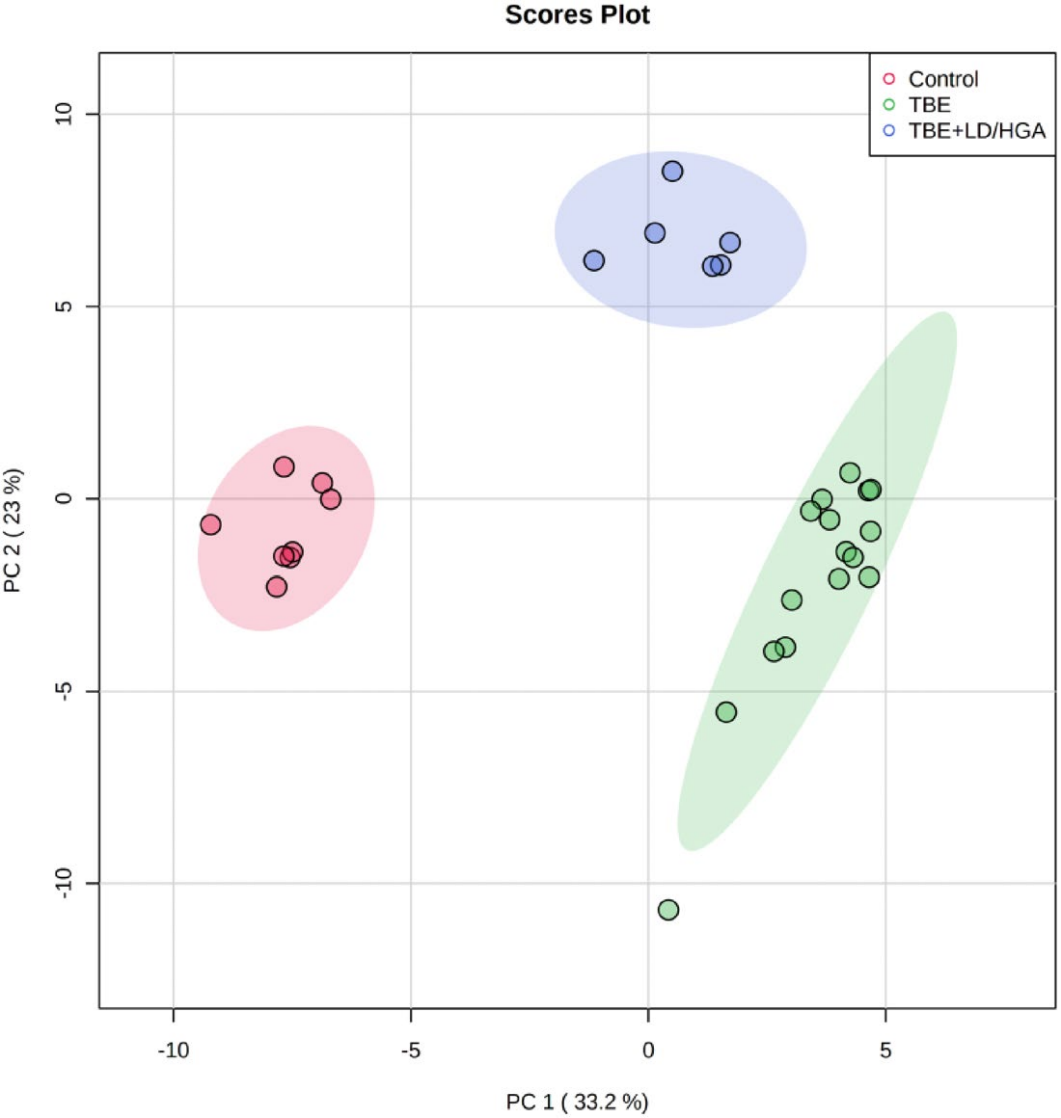
Two-dimensional principal component analysis (2D PCA) scores plot of the relative PL content in the plasma of healthy subjects (Control) and TBE patients (TBE) and TBE patients with co-infections (TBE + LD/HGA).

PCA analysis was used to confirm the quality of the datasets and to visualize differences between groups of samples in the unsupervised analysis. PCA score plot corresponding to PL data set (Fig. 1) demonstrated clear separation of three analyzed groups. The model captured 56.2% of the total variance. The first component PC1 (33,2%), which accounts for the greatest variation of the model, describes the discrimination between plasma samples from healthy subjects scattered in the left region of the plot and both groups of patients scattered in the right region. However, the second component PC2 (23%) is responsible for observed separation of co-infected TBE patient’s plasma samples from the other two groups (control and TBE).

**Figure 2:**
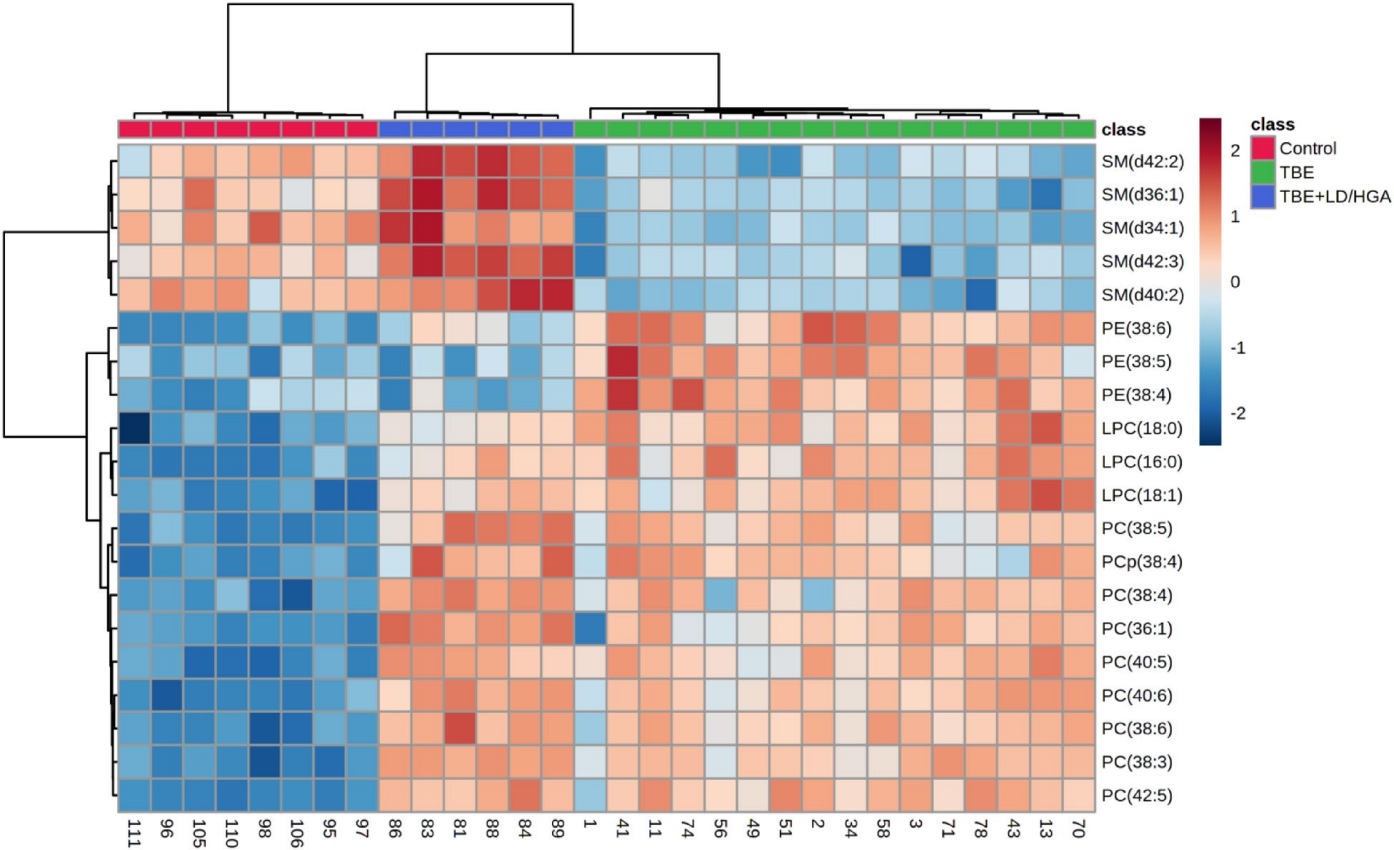
Two-dimensional hierarchical clustering heat map of the 20 most discriminating PL species (according to One-way ANOVA) identified in the plasma of healthy subjects (Control) and TBE patients (TBE) and TBE patients with co-infections (TBE + LD/HGA).

**Figure 3:**
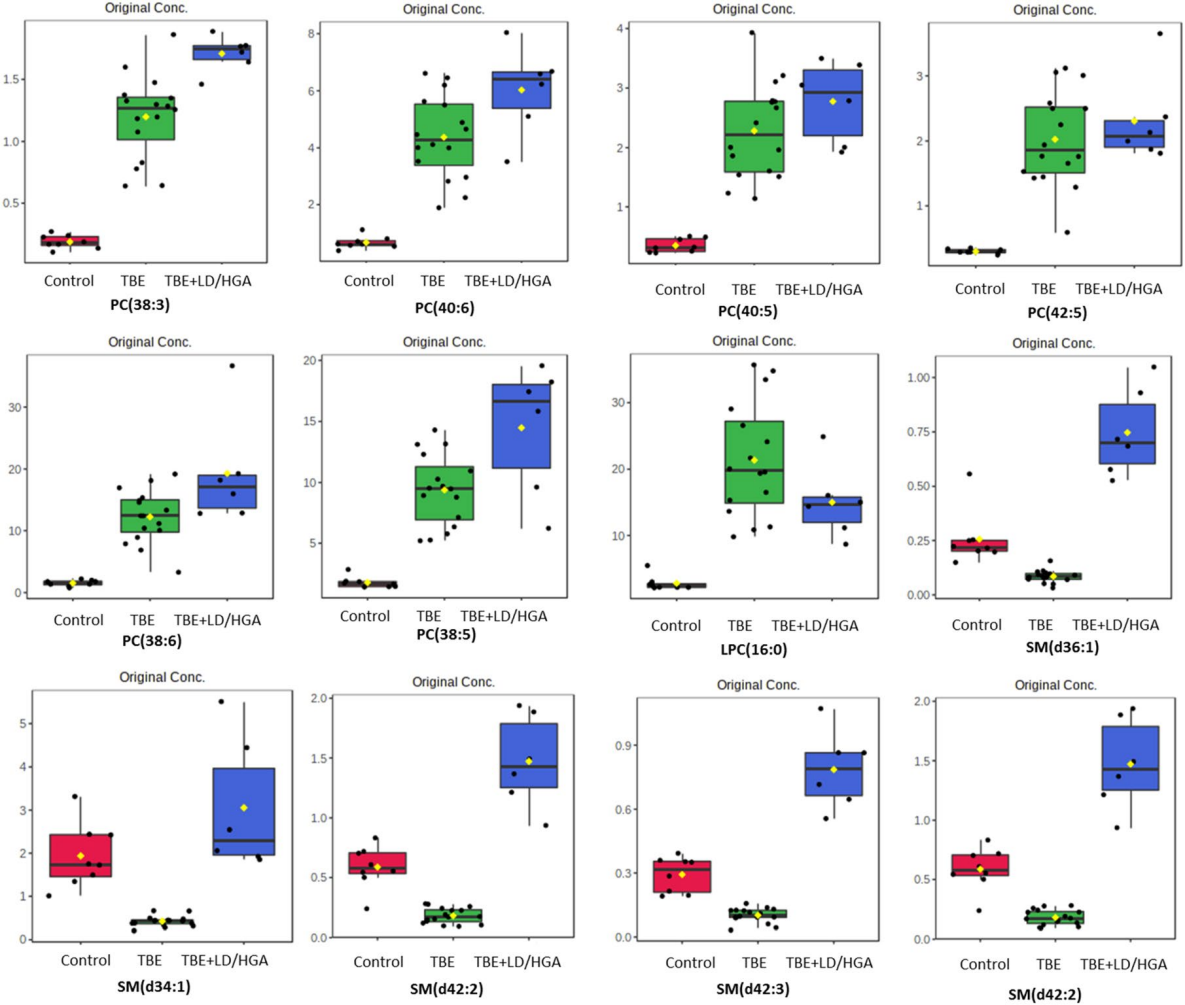
Relative abundances of differential (according to One-way ANOVA) PL species in the plasma samples collected from patients and healthy subjects. (red box: control group; blue box: TBE patients co-infected with LD/HGA; green box: TBE patients).

We also performed univariate analysis in order to evaluate the variation in the relative abundance of PL species belonging to each class. We sorted PL molecular species using the p values from the One-way ANOVA test (Table 3, Figs. 2, 3).

**Table 3:** The alteration observed in the molecular species of 20 most discriminating (according to One-way ANOVA) PL species from PC, LPC, PE and SM class in the plasma of healthy subjects (Control) and TBE patients (TBE) and TBE patients with co-infections (TBE + LD/HGA); mean difference was significant at the 0.05 level (p < 0.0001****; p < 0.001***; n.s.–not significant; the up arrows represent up-regulation and down arrows represent down-regulation.

PL class	PL specie	PL molecular specie	Log_2_ (fold-change)					
			TBE vs Control	*p*-value	TBE + LD/HGA vs Control	*p*-value	TBE + LD/HGA vs TBE	*p*-value
PC	PC(38:3)	PC(18:1/18:2)	2.66 ↑	****	3.18 ↑	****	–	ns
PC	PC(40:6)	PC(18:0/22:6)	2.71 ↑	****	3.16 ↑	****	–	ns
PC	PC(40:5)	PC(18:0/22:5)	2.70 ↑	****	2.99 ↑	****	–	ns
PC	PC(42:5)	PC(20:0/22:5)	2.75 ↑	****	2.94 ↑	****	–	ns
SM	SM(d36:1)	SM(d18:1/18:0)	1.59 ↓	****	1.55 ↑	****	3.14 ↑	****
SM	SM(d34:1)	SM(d18:1/16:0)	2.20 ↓	****	–	ns	2.85 ↑	****
PC	PC(38:6)	PC(16:0/22:6)	2.99 ↑	****	3.65 ↑	****	–	ns
SM	SM(d42:2)	SM(d18:1/24:1)	1.71 ↓	****	1.32 ↑	****	3.03 ↑	****
PC	PC(38:5)	PC(18:1/20:4)	2.40 ↑	****	3.02 ↑	****	–	ns
SM	SM(d42:3)	SM(d18:1/24:2)	1.54 ↓	****	1.43 ↑	****	2.96 ↑	****
LPC	LPC(16:0)	LPC(16:0)	2.92 ↑	****	2.41 ↑	****	–	ns
SM	SM(d40:2)	SM(d18:1/22:1)	2.06 ↓	****	1.07 ↑	***	3.13 ↑	****
PC	PC(38:4)	PC(18:0/20:4)	3.10 ↑	****	3.94 ↑	****	–	ns
LPC	LPC(18:0)	LPC(18:0)	3.41 ↑	****	2.35 ↑	****	1.06 ↓	***
LPC	LPC(18:1)	LPC(18:1)	3.63 ↑	****	3.12 ↑	****	–	ns
PC	PC(36:1)	PC(16:0/20:1)	2.11 ↑	****	2.88 ↑	****	–	ns
PE	PE(38:6)	PE(16:0/22:6)	3.20 ↑	****	1.65 ↑	****	1.55 ↓	****
PC	PCp(38:4)	PC(P-18:0/20:4)	1.97 ↑	****	2.31 ↑	****	–	ns
PE	PE(38:5)	PE(16:0/22:5)	2.16 ↑	****	–	ns	2.01 ↓	****
PE	PE(38:4)	PE(16:0/22:4)	1.99 ↑	****	–	ns	1.99 ↓	****

We observed statistically significant decrease of SM species, namely SM(d36:1), SM(d42:2), SM(d42:3) and SM(d40:2), in the plasma of TBE patients, when compared to control group (Fig. 2). In contrast, the relative content of these SM species in the plasma of TBE patients co-infected with bacteria was higher than in the plasma of healthy subjects. We also found that PC species, namely PC(38:3), PC(40:6), PC(40:5), PC(42:5), PC(38:6), PC(38:5), PC(38:4) and PC(36:1), were more abundant in the plasma of both groups of patients (TBE and TBE + LD/HGA), in comparison to the relative contents of these species in the plasma of control group. Moreover, observed up-regulation of PC in both groups of patients was accompanied by an increase in the relative levels of lyso-PC species LPC(16:0), LPC(18:0) and LPC(18:1). In addition to the above, observations up-regulation of certain PE species (PE(38:5), PE(38:4) and PE(38:6)) was found in the plasma of TBE patients (Fig. 2).

### Comparison of the plasma CER profile of healthy subjects (control) and TBE patients (TBE) and TBE patients with co-infections (TBE + LD/HGA)

We have identified CER species from two most abundant classes identified in examined plasma samples, namely CERs containing non-hydroxy fatty acids [N] and sphingosine [S] (CER[NS]) and CERs containing non-hydroxy fatty acids [N] and dihydrosphingosine [DS] (CER[NDS]) (Supplementary Table S4).

**Figure 4:**
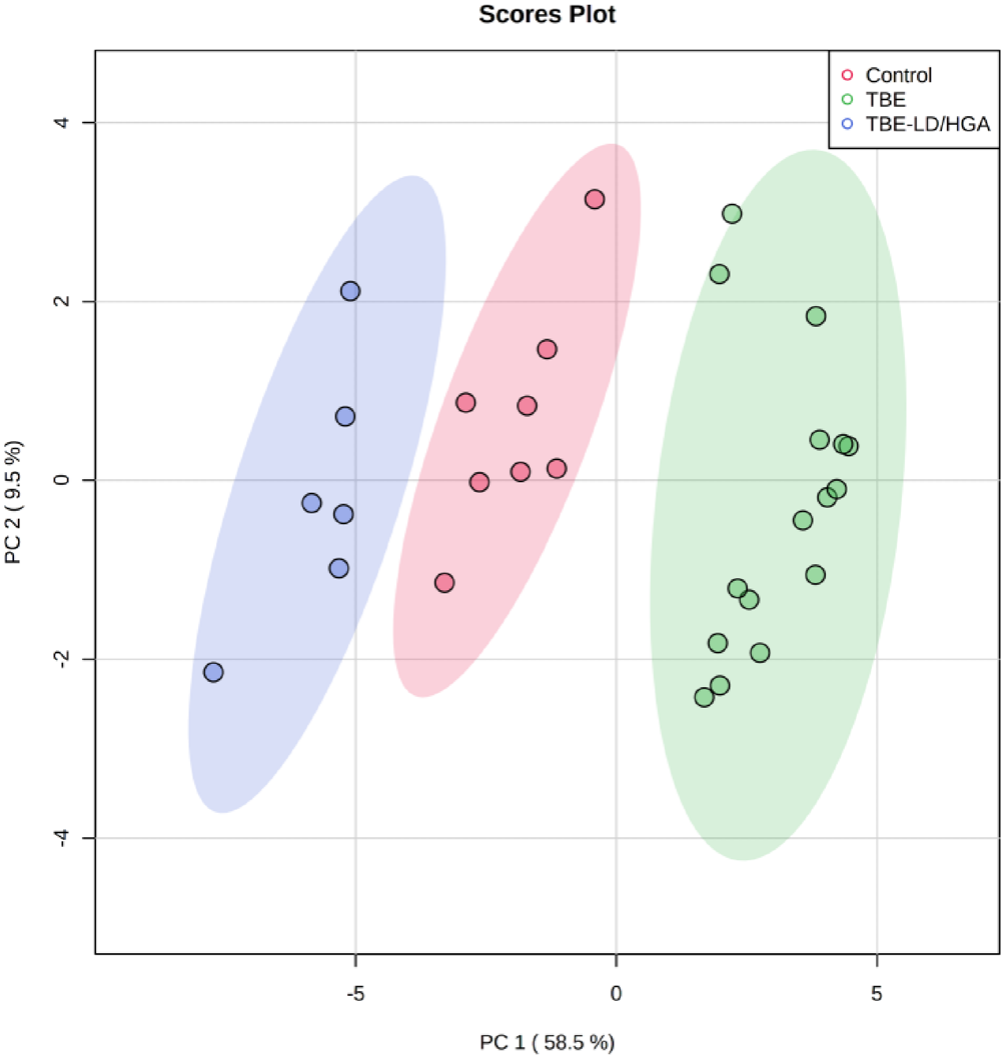
Two-dimensional principal component analysis (2D PCA) scores plot of the relative CER content in the plasma of healthy subjects (Control) and TBE patients (TBE) and TBE patients with co-infections (TBE + LD/HGA).

The 2D PCA scores plot corresponding to the data from CER profiling shows clear discrimination of the three analyzed groups of plasma samples. The model captured 68% of the total variance (Fig. 4) with the PC1 component (58.5%) and the second component PC2 accounted for 9.5%.

The variation between the groups is most pronounced along with the PC1 component (58.5%), which mainly describes the discrimination between plasma samples from TBE patients scattered in the right region of the plot and plasma samples from the other two groups (control and TBE patients co-infected with LD/HGA). The control group was scattered in the central region of the plot between both groups of patients (TBE and TBE + LD/HGA) indicating on an opposite direction of changes in the content of CER species, that has been confirmed by the univariate statistical analysis.

**Figure 5:**
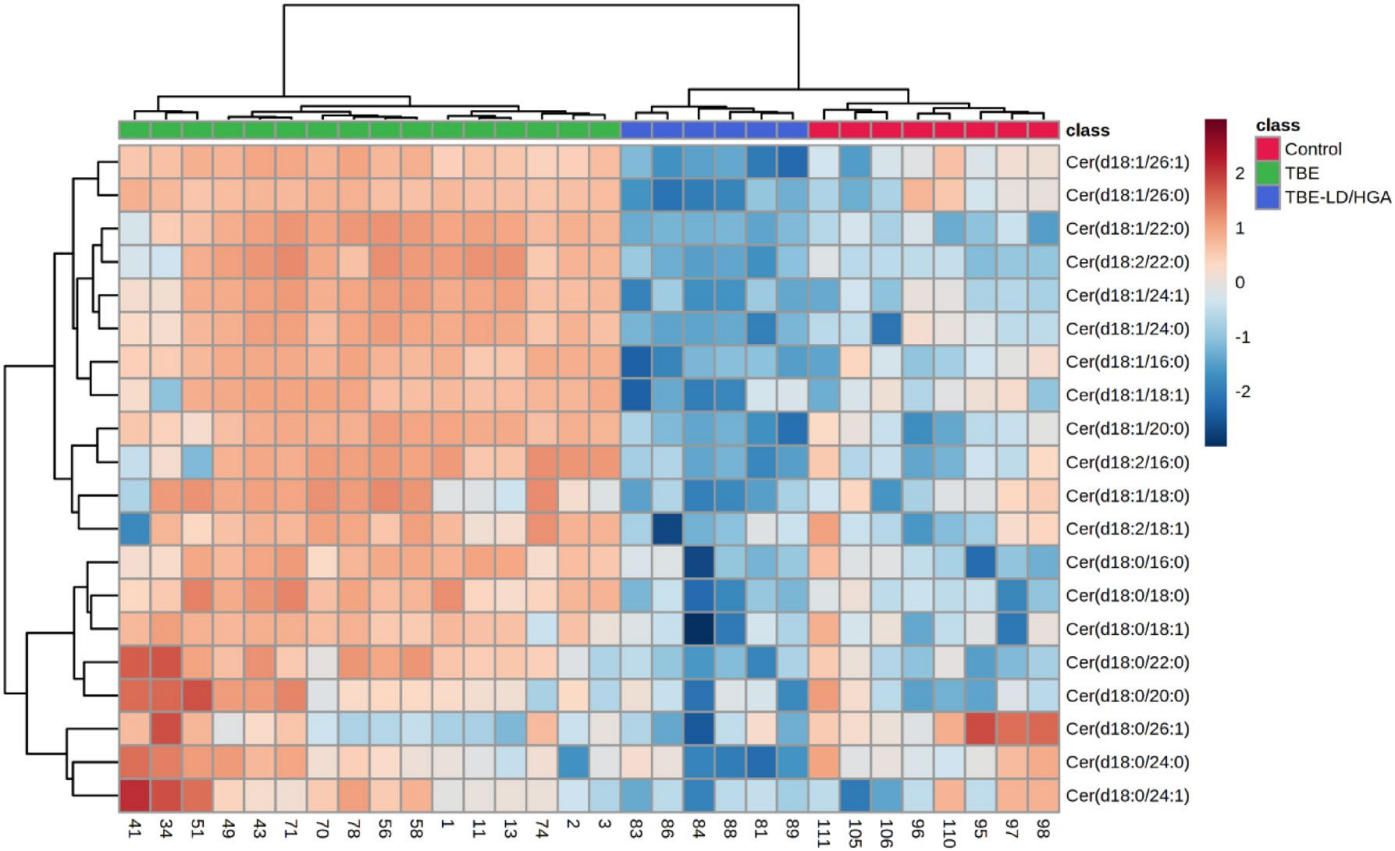
Two-dimensional hierarchical clustering heat map of the 20 most discriminating ceramide species (according to One-way ANOVA) identified in the plasma of healthy subjects (Control) and TBE patients (TBE) and TBE patients co-infected with LD (TBE + LD/HGA).

Obtained results also showed that changes in the SM content observed in the plasma of patients induced changes in the CERs relative levels. Univariate analysis of data set comprising CER species revealed an opposite direction of changes of their relative abundances in the plasma of two groups of patients (TBE and TBE + LD/HGA) in comparison to healthy subjects (Fig. 5). We found that almost all relevant CER species (Table 4) belonging to CER[NS] and CER[NDS] classes were up-regulated in the plasma of TBE patients, while general tendency to decrease of relative content of these CER species was observed in TBE + LD patients. Among all relevant CERs only one CER[NDS] CER specie, namely Cer(d18:0/26:1) was significantly down-regulated in the plasma of TBE patients.

**Table 4:** The alterations observed in the 20 most discriminating (according to the one-way ANOVA) CER species (CER[NS], CER[NDS]) in the plasma of healthy subjects (Control) and TBE patients (TBE) and TBE patients with co-infections (TBE + LD/HGA).

CER class	CER specie	Log_2_ (fold-change)					
		TBE vs Control	*p*-value	TBE + LD/HGA vs Control	*p*-value	TBE + LD/HGA vs TBE	*p*-value
CER[NS]	Cer(d18:1/22:0)	4.97 ↑	****	2.21 ↓	*	7.18 ↓	****
CER[NS]	Cer(d18:1/24:1)	5.28 ↑	****	3.33 ↓	****	8.62 ↓	****
CER[NS]	Cer(d18:1/26:1)	1.94 ↑	****	4.37 ↓	**	6.31 ↓	****
CER[NS]	Cer(d18:1/26:0)	1.43 ↑	****	3.77 ↓	**	5.20 ↓	****
CER[NS]	Cer(d18:1/16:0)	2.31 ↑	****	2.75 ↓	**	5.07 ↓	****
CER[NS]	Cer(d18:2/22:0)	4.56 ↑	****	1.97 ↓	**	6.53 ↓	****
CER[NS]	Cer(d18:1/24:0)	4.03 ↑	****	4.01 ↓	*	8.05 ↓	****
CER[NS]	Cer(d18:1/20:0)	2.46 ↑	****	2.14 ↓	*	4.60 ↓	****
CER[NDS]	Cer(d18:0/18:0)	2.42 ↑	****	1.21 ↓	*	3.63 ↓	****
CER[NS]	Cer(d18:1/18:1)	2.02 ↑	****	1.45 ↓	*	3.48 ↓	****
CER[NS]	Cer(d18:2/16:0)	1.83 ↑	****	1.50 ↓	*	3.34 ↓	****
CER[NDS]	Cer(d18:0/22:0)	2.28 ↑	****	1.29 ↓	*	3.56 ↓	****
CER[NDS]	Cer(d18:0/16:0)	2.17 ↑	****	–	ns	3.14 ↓	****
CER[NS]	Cer(d18:1/18:0)	1.67 ↑	*	2.41 ↓	**	4.08 ↓	****
CER[NDS]	Cer(d18:0/18:1)	1.41 ↑	****	1.14 ↓	*	2.56 ↓	****
CER[NS]	Cer(d18:2/18:1)	–	ns	–	ns	1.94 ↓	***
CER[NDS]	Cer(d18:0/24:0)	–	ns	2.30 ↓	**	2.98 ↓	**
CER[NDS]	Cer(d18:0/26:1)	1.19 ↓	*	2.83 ↓	*	1.63 ↓	*
CER[NDS]	Cer(d18:0/20:0)	1.86 ↑	**	–	ns	2.67 ↓	**
CER[NDS]	Cer(d18:0/24:1)	1.82 ↑	*	2.56 ↓	*	4.39 ↓	***

All the alterations are significant at the P < 0.05 level. *(non-hydroxy fatty acid [N], α-hydroxy fatty acid [A], dihydrosphingosine [DS], sphingosine [S]);* mean difference was significant at the 0.05 level (p < 0.0001****; p < 0.001***; p < 0.01**; p < 0.05*; n.s. – not significant; the up arrows represent up-regulation and down arrows represent down-regulation.

**Figure 6:**
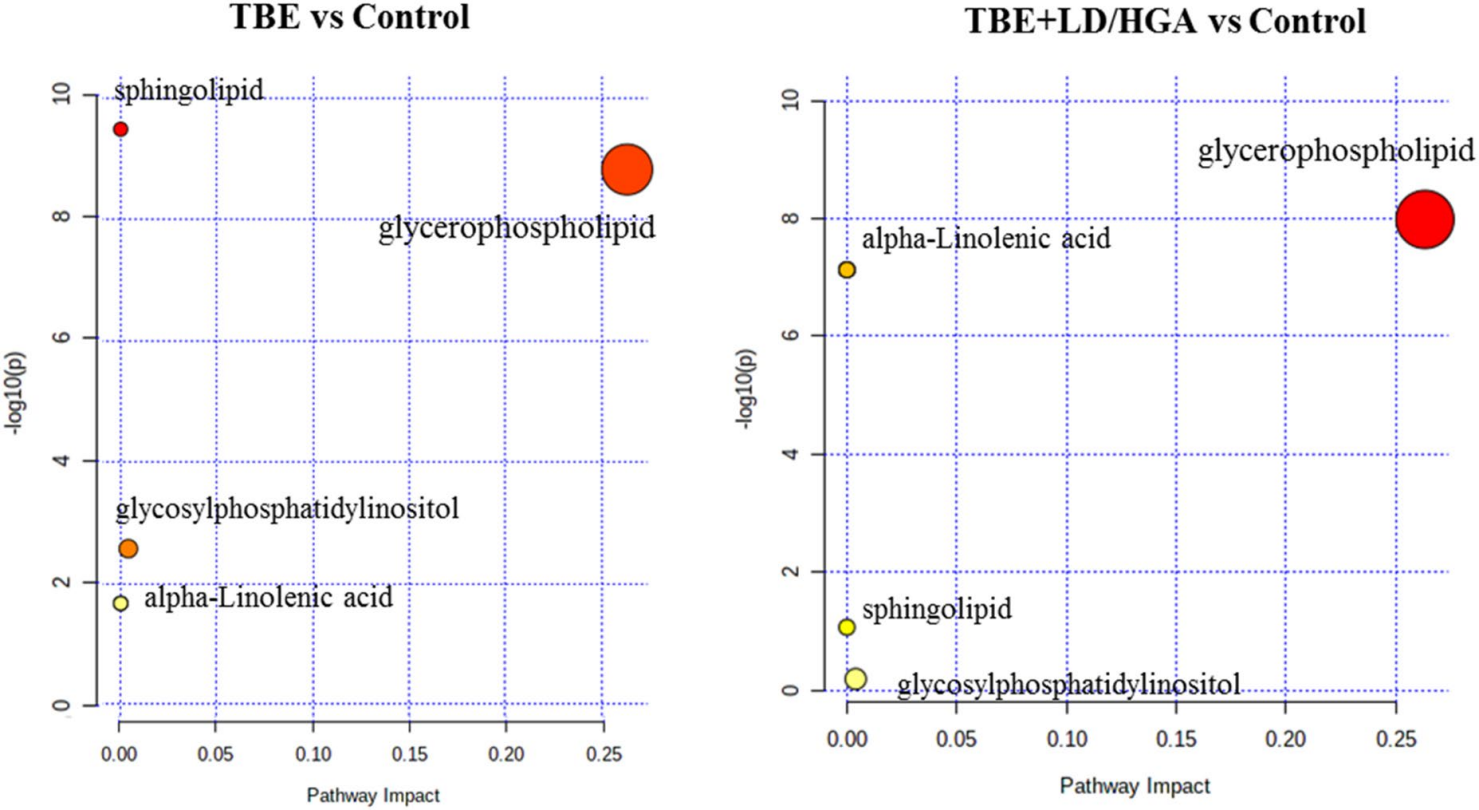
Graphical representation of the pathway analysis of TBE patients vs healthy subjects; left panel pathway analysis of differentiating lipids in the TBE vs control group, right panel pathway analysis of differentiating lipids in the TBE + LD/HGA vs control group. The gradual color of each circle reperesents the *p*-value (the lower p-value the color changes from yellow to red), while the size of each circle represents pathway impact (a combination of the centrality and number of PL species enriched in the pathway).

Finally, we performed pathway analysis, by subjecting differentiating PLs to the Kyoto Encyclopedia of Genes and Genome (KEGG) database via the Metaboanalyst online website. A graphical representation of all matched pathways according to *p*-values is shown in the Fig. 6. The results of the pathway analysis revealed differences in lipid metabolism between TBE and TBE + LD/HGA patients. Differentiating PL species were more significantly enriched in the LA metabolism, AA metabolism and sphingolipid metabolic signaling pathways in the TBE group when compared to TBE + LD/HGA group (P < 0.05) (Supplementary Table S5). However, differential PLs were to the similar extent enriched in glycerophospholipid metabolism both in TBE patients and TBE patients co-infected with bacteria (Fig. 6).

**Figure 7:**
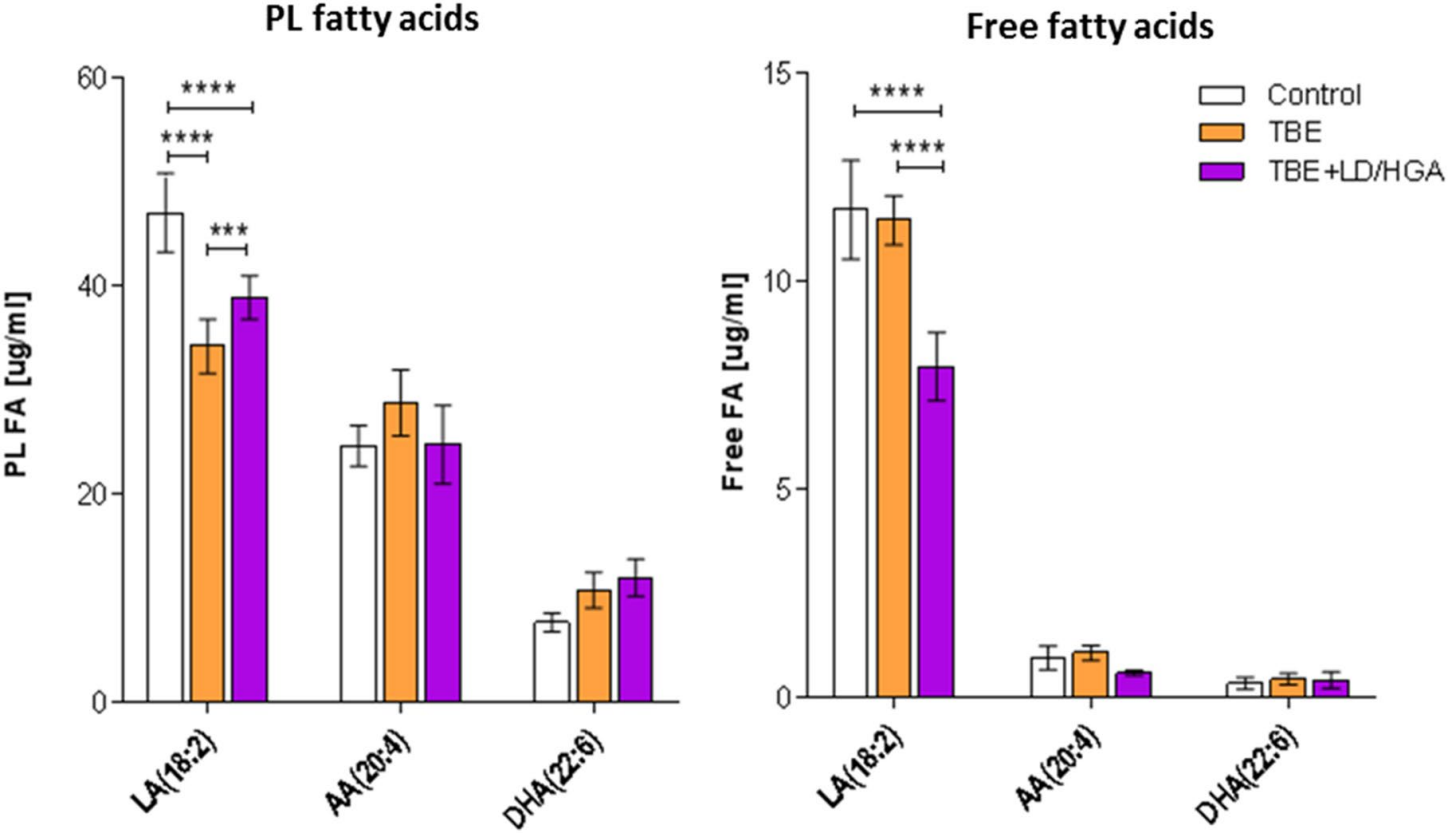
The levels of arachidonic (AA), linoleic (LA) and docosahexaenoic acid (DHA) in the plasma of healthy subjects (Control), TBE patients (TBE) and TBE patients co-infected with LD and HGA; left panel PL fatty acids, right panel free fatty acids, ***p < 0.001, ****p < 0.0001.

We also quantified fatty acids by GC-FID in all groups of plasma samples examined in the study (Fig. 7). Obtained results showed that the levels of free and PL linoleic acid (LA) were significantly lower in the plasma of both TBE and TBE-bacteria-coinfected patients in comparison to their levels in healthy people, while decrease in the level of free LA was observed only in TBE patients co-infected with and *B. burgdorferi* or *A. phagocytophilum.* In the case of PL docosahexaenoic acid (DHA) and arachidonic acid (AA), an opposite direction of changes was observed. Total plasma level of PL AA was elevated only in TBE patients, while an increase in the level of PL DHA was also observed in co-infected TBE patients when compared to healthy subjects (Fig. 7).

## Discussion

It is well known that viruses have developed numerous strategies to take control of essential cellular factors and functions. Enveloped viruses use their membrane PLs to enable viral apoptotic mimicry, a process in which viruses due to external exposure of PS on the viral membrane, can easily enter into host macrophages through phagocytosis ^22^. However, during viral infection, viruses rely on the host cells to reproduce. They reprogram cellular metabolism to produce macromolecules required for virion replication and assembly^23^. It is well known that there are strong relationships between flavivirus infections and host cell, including reprograming PL synthesis and metabolism to create a favorable environment for viral multiplication^24^. PLs of the host cells have been also shown as critical to viral replication^25^. Importantly, the flaviviruses, the family of viruses which includes the TBEV, by formation of replication complexes with proteins of the host promote PL synthesis leading to their extensive rearrangement in cell membrane^24^. In addition, more recently it has been revealed that PLs, as reservoir of fatty acids, may be involved in viral attachment and penetration of the host organism^26^. Therefore, the assessment of the host PL metabolism seems to be very important in the context of TBEV infection.

However, so far, only single metabolomics study has been presented to identify metabolites involved in the acute inflammatory response or immune regulation in TBEV infection, but the exact mechanisms of metabolic changes, especially in lipids, are still not fully understood^3,27^. This is especially true for cases of co-infections with bacteria transmitted simultaneously with TBEV by ticks.

Results of the present study indicate that plasma PLs show significant variation between healthy subjects and two groups of TBE patients. We found increased relative content of phosphatidylcholines (PCs) in the plasma of TBE patients and patients co-infected with bacteria. PCs constitute about 50% of cellular membrane PLs, therefore they are considered as the main reservoir of PUFAs^28^. The results of the metabolic pathway analysis obtained in this study confirmed that the LA (18: 2, ω-6) and AA (20: 4, ω-6) pathways are modified in both groups of patients (TBE and TBE + LD/HGA). This observation is supported by quantitative fatty acids analyses which revealed significant elevation of the level of PL AA in the plasma of TBE patients analyzed in the study. These is in partial accordance with the results of PL profiling which showed that PC containing AA, namely PC(18:1/20:4) and PC(18:0/20:4) were more abundant in the plasma of TBE patients and TBE + LD/HGA patients than in the plasma of healthy subjects. In contrast to AA, the level of total LA in PL fraction was significantly lower in the plasma of TBE patients and co-infected patients when compared to healthy subjects. Interestingly, significant decrease of free LA level was observed only in the plasma of TBE patients with coinfections. It is well known that PL bearing LA and AA are the main source of 4-HNE and isoprostanes, which are considered to be one of the major end products of lipid peroxidation^29,30^. Increased levels of isoprostanes, free 4-HNE and adducts of 4-HNE with protein, were found not only in the plasma of TBE patients but also in the plasma of subjects infected by *Borrelia burgdorferi,* the main cause of LD^6,31^.

Moreover, it is well known that free PUFAs are involved in the regulation of inflammatory processes as precursors of eicosanoids. Overall, eicosanoids derived from AA and LA are pro-inflammatory and promote an inflammatory response aimed at eliminating the pathogen with minimal damage to the host organism^32^. Furthermore, PUFAs, in particular AA and LA, are known to be involved in antiviral activity through various mechanisms^33^. Fatty acid synthesis has been shown to be related to viral replication^24^. In particular, PUFAs have antimicrobial properties, mainly by targeting microbial cell membranes, generating free radicals and formation of cytotoxic lipid peroxides or bioactive immuno-modulating metabolites^34^. Therefore, the above mentioned effects may to some extent explain observed in this study increase in the amount of PCs containing AA and LA in the plasma of TBE patients but also patients co-infected with bacteria. Enhanced PC synthesis has also been found during infection with various viruses including Dengue virus^35^, Flock House virus^36^, and poliovirus^37^. Results of recent metabolomic study also confirmed up-regulation of PC species in the serum of TBE patients^3^. It has been shown that flaviviruses stimulate host PCs synthesis at viral replication sites^38^. In addition, it was also found that poliovirus promotes the import of fatty acids mainly incorporated into PCs to the viral replication sites^37^. Considering above, it may be suggested that increased content of PC containing AA and LA is required in order to provide appropriate response to TBE infection and co-infection with bacteria since both PUFAs have been shown to possess antimicrobial action (against gram-negative, as well as gram-positive bacteria) and have been suggested to inactivate enveloped viruses in the passivation process^5,39^.

An increase in PC content observed in our study was accompanied by up-regulation of LPC species in the plasma of both TBE and bacterial-coinfected patient groups. The literature reports indicate that viral infections cause increase in the activity of several enzymes critical to PL biosynthesis and metabolism, including phospholipase A2 responsible for the formation of lysophospholipids, which have also been suggested to be essential for flavivirus replication^40^. Another enzyme that hydrolyzes the long-chain PUFAs at the sn-2 position of the glycerol backbones of PLs is PAF-AH. Significantly increased PAF-AH activity has been previously reported in the plasma of patients with TBE^6^. Since the specific substrates for PAF-AH include complex oxidized PLs, such as isoprostanes—containing phosphatidylcholines^41^, this enzyme can be considered the main factor stimulating the increase in free isoprostanes production observed in the plasma of TBE patients^6^. Considering the above and the up-regulation of LPC species observed in the present study, accompanied by increased synthesis of PC containing PUFAs, it can be assumed that PL hydrolysis is also crucial in TBEV replication. Therefore, this specific host membrane PL rearrangement most likely can be used by TBEV for replication.

Although the directions of changes in the above-mentioned classes of PLs are similar in the plasma of patients with TBEV and those infected with TBEV and bacteria, our study identified a different regulation of PL content that determines the discrimination between the two groups of patients. Firstly, we found that SM species were less abundant in the plasma of TBE patients compared to healthy subjects, while the plasma SM content of TBE patients co-infected with bacteria was higher than that of healthy subjects. This was reflected by the pathway analysis which showed that sphingolipid metabolic signaling pathway had different enrichment in SM species in the TBE group compared to the group of patients with co-infection. So far, SM has been shown to play a key role in virus formation and pathogenesis^24^. This may explain the increase in plasma levels of SM species in TBE patients observed in this study. However, the observed differential regulation of SM depending on the absence or existence of co-infection may be related to the demyelination of neurons, as previously suggested in studies on neuroborreliosis^42^. It has been suggested that *B. burgdorferi* can induce an autoimmune response by acting on myelin sheaths, since the major component of myelin glycolipid galactocerebroside, has structural similarities to the *B. burgdorferi* glycolipid antigen BbGL-2^43^. As a consequence, in co-infected patients, SM synthesis may be induced by activation of the SM-CER pathway as indicated by the changes in CER content identified in this study. We found that almost all relevant CERs species belonging to CER[NS] and CER[NDS] classes were up-regulated in the plasma of TBE patients, while general tendency to decrease of relative content of these CER species was observed in co-infected TBE patients. The observed increase in the CER content may explain the reduction in the level of SM in patients with TBE. In addition, a significant decrease in the relative content of SM in the plasma of TBE patients, accompanied by an increase in CERs, may indicate their participation in the propagation of infection with TBE virus. Previously, it has been also suggested that increased activity of sphingomyelinase, the enzyme responsible for the hydrolysis of SM to CER, leads to increased CER synthesis accompanied by a dramatically enhanced infection with Japanese encephalitis virus, and its progression^44^.

The second class of PLs that distinguish TBE patients from co-infected TBE patients is PE. This study showed that some PE species are up-regulated in plasma of TBE patients, while plasma levels of PE in patients co-infected with bacteria are similar to that in healthy subjects. So far, it has been found that PEs are involved in the entry of flaviviruses^24^. On the other hand, it is known that the accumulation of PE in the host cells results in a significant inhibition of the inflammatory responses induced by flaviviruses, including the dengue virus^45^. These effects may serve as a possible explanation for the increased levels of PE species in the plasma of patients with TBE observed in this study, especially since PE are considered to be highly anti-inflammatory lipids that can reduce inflammation caused by both bacteria and viruses^45^. On the other hand, the lack of significant differences between the PE content in the plasma of TBE patients co-infected with bacteria and healthy people can be explained by the fact that, as previously suggested in the literature, *Borrelia* spp. inhibits viral replication in individuals susceptible to tick-borne encephalitis^46^. Nevertheless, this problem is still not fully understood.

## Conclusion

Results of this study show differences in the plasma PL profile of patients with TBE and patients with TBE co-infected with bacteria (*B. burgdorferi* or *A. phagocytophilum*). At the same time, it should be emphasized that the differences in the lipidomic signature concerning PE, SM and CER species distinguish TBE patients from TBE patients co-infected with bacteria and may help to better understand the complex pathomechanisms resulting from viral and bacterial co-infection. Moreover, the opposite direction of changes in SM and CER contents may be useful in development of diagnostic tools allowing for the differentiation of patients with tick-borne encephalitis from patients with TBE co-infected with bacteria. We also believe that drugs, which may modify CER metabolism by modulation of sphingomyelinase activity or by promoting de novo CER synthesis, may contribute to the effective pharmacotherapy of TBE, also in the case of co-infection with bacteria.

## Supplementary Information

Supplementary Information 1.

Supplementary Information 2.

Supplementary Information 3.

## Data Availability

The datasets generated during and/or analyzed during the current study are available online as supplementary material.
