# Supplementary material for: Differences in the plasma phospholipid profile of patients infected with tick-borne encephalitis virus and co-infected with bacteria
Source: Sci Rep. 2022 Jun 9;12:9538. doi: 10.1038/s41598-022-13765-2 (PMC9184562; doi:10.1038/s41598-022-13765-2)
Supplement: Supplementary file 1 — Supplementary Information 1. [file 41598_2022_13765_MOESM1_ESM.pdf]

Table S1. Relative content (related to internal standard) of each phospholipid species identified in the plasma of healthy subjects (Control), TBE patients (TBE) and TBE patients co-infected with Lyme disease and Anaplasmosis (TBE+LD/HGA). Data obtained using MZmine software (XLSX).

	A	B	C	D	E	F	G	H	I	J	K	L	M
1	Name	1	2	3	11	13	34	41	43	49	51	56	58
2	Label	TBE	TBE	TBE	TBE	TBE	TBE	TBE	TBE	TBE	TBE	TBE	TBE
3	PC(34:2)	2.925332	33.80223	30.19966	23.29023	27.74852	15.04441	24.47336	33.99774	19.45819	40.29418	7.844334	25.28279
4	PC(36:2)	4.855632	11.69079	19.77239	12.6666	26.32963	8.088657	15.59126	20.6425	14.18827	21.25064	10.10985	16.48725
5	PC(34:1)	2.41753	34.39251	36.04261	21.27034	21.44267	12.50117	31.98494	28.8929	8.669419	28.63791	6.12257	21.73468
6	PC(36:4)	11.59523	46.12468	30.60919	39.27478	28.78208	10.07764	39.33928	22.42554	21.18906	31.83056	11.04526	17.37441
7	PC(36:3)	4.653938	9.763406	16.73069	15.00817	9.933694	5.341821	13.68158	8.687195	10.19764	15.24735	6.291842	7.605247
8	PC(38:4)	10.03901	22.95007	17.10598	20.61491	18.91392	15.17011	30.95805	25.04989	11.13039	25.35922	11.37108	15.30665
9	PC(38:6)	3.290567	15.36729	14.9245	18.1445	14.61688	7.884576	12.38492	13.30424	10.3981	10.05107	6.868699	19.18962
10	PC(36:1)	1.148661	6.256417	3.133197	3.690427	6.09589	3.450299	3.85737	4.267748	1.934026	2.333306	2.200526	3.223671
11	PC(38:5)	5.200495	13.12468	13.1611	12.30367	9.474112	8.929172	14.30421	9.540804	8.791559	10.95621	6.335658	7.115789
12	PCp(38:8)	1.074947	2.31904	4.101463	2.239134	3.185609	2.975393	3.579914	3.526488	2.342842	3.676631	1.985821	2.869545
13	PC(38:3)	1.883735	4.390906	4.258837	6.722231	5.181661	3.892332	5.143466	6.604397	3.765771	6.209273	2.306635	1.982059
14	PC(40:6)	1.890452	4.116689	3.527497	5.500723	6.453879	2.819117	4.463713	6.610372	2.956983	4.889177	2.244332	4.65728
15	PC(34:0)	0.332447	4.07265	1.52198	3.041685	3.045674	1.421057	1.598228	2.013473	1.706981	4.04356	0.893026	2.336592
16	PCp(40:10	0.660855	0.287191	2.767354	2.768477	1.509691	0.970073	1.522175	1.526403	1.655001	0.992168	0.255372	1.421894
17	PC(36:5)	0.863378	1.335118	2.808934	1.650721	1.695904	2.346452	1.774176	1.260516	0.697668	1.79578	1.028747	1.478739
18	PCp(46:11	0.385056	1.73047	1.448717	3.322575	3.084631	0.740091	2.382835	1.692144	1.708505	2.244323	0.757906	1.867224
19	PCp(38:4)	0.744793	1.60437	1.200208	1.972731	2.005699	1.451378	2.255119	0.660786	1.540946	1.582814	1.223689	1.382349
20	PC(40:5)	1.537866	3.107026	2.787671	2.41632	3.930879	1.507949	3.210261	2.668599	1.138089	1.229172	1.601095	1.861904
21	PCp(44:11	0.593965	2.497715	2.580932	3.005597	1.939959	1.444435	1.767524	2.499131	1.285897	3.117228	1.526841	2.249612
22	PC(32:1)	0.016309	1.197185	0.871481	0.638524	0.971443	0.252058	0.893959	0.412332	0.224431	0.627534	0.013339	0.367325
23	PCp(42:9)	0.202469	2.080115	2.892422	2.877408	2.147308	0.967185	2.41141	1.851581	1.070214	2.300302	0.844628	1.425467
24	PCp(38:7)	0.311424	1.837537	2.645736	2.569387	2.354544	1.542117	1.865091	1.858241	1.163912	1.588733	1.022204	1.841679
25	PCp(40:9)	0.638908	1.076419	1.474675	1.37462	1.297903	0.777525	1.256895	1.284553	1.196998	1.183922	0.64243	0.829394
26	LPC(16:0)	15.33825	29.05495	19.57173	9.799265	26.58075	19.38861	33.45727	34.77116	13.62583	10.84679	35.67675	20.06508
27	LPC(18:2)	3.58309	4.570455	4.120368	1.752201	11.29349	9.774479	8.937963	3.899859	4.214297	3.950719	8.326846	8.854728
28	LPC(18:1)	2.288226	3.32336	2.972676	1.103757	9.524628	4.369749	3.87155	6.529593	1.90338	3.029485	4.074487	4.438366
29	LPC(18:0)	5.010459	1.972412	5.552362	2.421868	9.976244	3.992812	7.068618	7.495936	4.671821	6.139677	4.700202	2.7162
30	PE(28:0)	3.84596	5.326147	3.989329	4.220045	4.595536	2.451794	3.346349	3.575168	3.179105	2.747736	4.36587	2.919888
31	PEo(38:5)/	1.881453	1.544859	0.776188	0.952362	2.546195	1.503877	0.631896	2.213522	2.046928	1.720906	2.025397	1.632607
32	PE(36:2)	0.258206	0.581569	0.24891	1.316052	0.427952	0.980088	0.815682	1.462211	0.456135	1.038664	0.717841	1.478333
33	PEo(38:6)/	0.917575	0.646187	0.897755	1.59569	0.892148	0.404334	0.91918	0.497036	0.950836	0.892904	0.85289	1.01038
34	PE(38:4)	1.372827	1.075112	1.103808	1.561572	1.024891	0.905929	2.939816	1.995803	1.173054	1.816894	1.405916	1.477991
35	PEo(36:5)/	0.789856	0.454243	0.271826	0.442919	0.482534	0.912984	0.539149	0.677338	0.721169	0.206835	0.319683	0.916811
36	PEo(38:7)/	0.62011	0.819939	0.741855	0.395195	0.846127	0.613283	0.589872	0.415572	0.895915	0.699062	0.179158	0.31675
37	PEo(36:3)/	0.600027	0.233731	0.134941	0.295398	0.222013	0.501323	0.404787	0.589873	0.192184	0.19175	0.348082	0.505928
38	PEo(36:4)/	0.055442	0.236553	0.184718	0.38628	0.2267	0.264704	0.601819	0.656452	0.130851	0.633954	0.351702	0.23025
39	PE(38:6)	0.443166	1.499982	0.556955	1.241096	0.928374	1.352375	1.246713	0.618503	0.408326	0.721981	0.316597	1.077529
40	PE(40:6)	0.38364	0.52267	0.385687	0.595929	0.409753	0.442729	0.721554	0.670639	0.270981	0.219897	0.285048	0.585671
41	PE(38:5)	0.326632	0.687469	0.461206	0.723859	0.414785	0.723202	1.187629	0.578787	0.412272	0.516858	0.644163	0.518089
42	[illegible]	[illegible]	[illegible]	[illegible]	[illegible]	[illegible]	[illegible]	[illegible]	[illegible]	[illegible]	[illegible]	[illegible]	[illegible]

	A	N	O	P	Q	R	S	T	U	V	W	X	Y	Z
1	Name	70	71	74	78	86	81	83	84	88	89	95	96	
2	Label	TBE	TBE	TBE	TBE	TBE+coinf	TBE+coinf	TBE+coinf	TBE+coinf	TBE+coinf	TBE+coinf	Control	Control	Contr
3	PC(34:2)	31.96274	24.11761	32.47767	49.74336	44.45411	52.67229	50.98965	39.57763	72.54984	44.15514	8.099544	11.78838	10.45
4	PC(36:2)	13.38895	11.47658	24.45163	29.66882	39.2473	23.69938	40.35183	40.35993	40.23606	32.88586	8.714604	7.606157	5.961
5	PC(34:1)	16.15374	25.53973	35.11695	27.85613	29.62441	46.39707	30.73556	66.87366	51.00737	37.87613	9.504667	6.064007	10.45
6	PC(36:4)	15.6442	39.90448	37.61619	49.70244	23.29868	29.754	38.16137	30.12074	47.47976	37.33086	7.838726	5.191233	8.068
7	PC(36:3)	7.477451	7.25442	15.51002	10.93802	12.95587	12.4256	21.11708	20.02378	12.42105	13.22595	4.365415	5.272886	1.943
8	PC(38:4)	11.7139	23.56613	26.07933	15.37293	13.03021	26.89142	20.81944	18.52724	35.06299	21.76706	4.756914	2.584594	4.242
9	PC(38:6)	16.97593	8.909556	12.39587	11.14608	12.85493	36.66255	15.99746	19.26764	12.78755	18.20381	2.225249	1.351012	1.778
10	PC(36:1)	4.137894	3.372656	3.140428	3.258247	9.853206	9.931557	3.687091	9.123403	11.41478	3.441747	1.203777	1.612372	0.832
11	PC(38:5)	9.685045	5.258137	10.27684	5.767886	6.222374	19.56217	9.624298	15.82949	17.43191	18.22877	1.724097	2.849588	1.851
12	PCp(38:8)	3.182359	4.69314	4.212929	2.852414	4.078543	3.817677	4.634941	0.431131	2.94643	5.459857	0.487634	0.516216	0.577
13	PC(38:3)	4.850958	3.160607	5.499372	3.156842	8.660234	6.230024	8.157706	4.189692	6.394338	8.129195	1.521565	1.030445	1.204
14	PC(40:6)	6.196908	4.008731	4.000389	5.625317	3.514891	8.039566	6.678199	6.230439	5.103978	6.59377	0.805289	0.386783	1.12
15	PC(34:0)	2.546629	1.210144	3.839825	4.966501	4.355926	4.800996	3.716834	6.373027	5.497963	3.608651	0.914407	0.665964	1.08
16	PCp(40:10	1.895319	1.712306	1.896121	1.93549	2.022864	3.474051	2.846132	2.78159	2.18378	2.55998	0.20762	0.201877	0.185
17	PC(36:5)	2.870184	1.05888	2.201669	1.356377	2.920208	4.603325	1.635904	1.7903	2.072	2.447464	0.416229	0.25329	1.094
18	PCp(46:11	1.604259	1.380052	2.381295	1.279698	2.221113	2.693158	2.171334	3.3189	2.978936	3.008668	0.469014	0.226945	0.337
19	PCp(38:4)	1.664815	0.929203	1.887056	0.846908	0.787618	1.680592	2.797267	1.491826	1.510252	2.625229	0.469267	0.363625	0.337
20	PC(40:5)	2.762087	2.005157	1.961794	2.774806	3.497879	3.049569	3.388043	2.005442	2.786642	1.924702	0.504934	0.454335	0.309
21	PCp(44:11	1.656815	1.426079	1.762189	3.053815	2.131775	1.812111	1.872633	3.646983	2.370981	1.998422	0.272064	0.293185	0.354
22	PC(32:1)	1.092461	0.798788	0.929674	0.301011	0.719875	1.285157	0.727872	1.000392	2.618027	1.350107	0.204898	0.076178	0.412
23	PCp(42:9)	2.059706	2.178029	1.631251	2.799421	1.846029	2.848399	2.352837	2.640894	3.017752	1.733358	0.490045	0.393478	0.367
24	PCp(38:7)	1.949056	2.362367	1.096762	1.611623	3.655232	2.188947	3.164619	2.473277	2.800574	3.366946	0.420102	0.444994	0.315
25	PCp(40:9)	1.349422	1.860642	1.326659	1.59902	1.719262	1.460993	1.772825	1.639654	1.886294	1.766429	0.137728	0.168825	0.22
26	LPC(16:0)	24.12792	11.29183	16.52488	21.67743	8.687832	15.04369	11.1526	14.37552	24.88947	16.06504	5.482681	2.198272	2.654
27	LPC(18:2)	7.166002	1.457105	2.322948	4.955476	2.116304	1.892701	3.562467	5.945444	3.955777	1.4475	1.082851	0.775826	1.309
28	LPC(18:1)	6.43875	1.871558	1.747256	2.542246	1.719519	1.552115	2.463742	3.795088	3.257263	3.093366	0.168023	0.497939	0.163
29	LPC(18:0)	4.919187	2.360622	2.493914	3.274316	2.00832	1.920084	1.570886	2.801836	2.288423	2.817734	0.461369	0.424554	0.658
30	PE(28:0)	2.819677	3.38836	4.165773	3.256864	1.296618	0.792759	1.212695	0.92945	0.810038	0.791948	1.584026	1.444146	1.017
31	PEo(38:5)/	2.144299	1.027108	2.073178	1.7868	0.288913	0.420888	0.358864	0.445379	0.1927	0.326022	0.519145	1.183353	0.919
32	PE(36:2)	1.208798	0.413564	0.919295	0.839227	0.207803	0.302409	0.624551	0.212803	0.360624	0.148671	0.263864	0.232151	0.426
33	PEo(38:6)/	0.883845	0.966957	1.405167	0.559964	0.128666	0.42495	0.235312	0.306994	0.160333	0.27447	0.223207	0.842149	0.856
34	PE(38:4)	1.267882	0.891889	2.439009	1.372663	0.198489	0.297773	0.721805	0.299834	0.252618	0.463886	0.487374	0.222836	0.54
35	PEo(36:5)/	0.359563	0.567318	0.625398	0.639939	0.078145	0.373839	0.075988	0.194305	0.139782	0.138639	0.268303	0.255398	0.545
36	PEo(38:7)/	0.842834	0.424674	0.747214	0.828798	0.161238	0.350027	0.162416	0.06099	0.104406	0.15931	0.436296	0.103045	0.533
37	PEo(36:3)/	0.452375	0.323966	0.314572	0.533122	0.063624	0.13783	0.067685	0.13939	0.12776	0.092785	0.21859	0.17029	0.157
38	PEo(36:4)/	0.426373	0.148054	0.381149	0.367525	0.048009	0.154051	0.10609	0.177832	0.078181	0.043069	0.180742	0.210938	0.197
39	PE(38:6)	0.859922	0.486454	0.98227	0.458421	0.169541	0.388508	0.472835	0.141429	0.317518	0.204114	0.12994	0.069772	0.07
40	PE(40:6)	0.629925	0.157952	0.477149	0.527694	0.071029	0.179214	0.162163	0.121887	0.17785	0.150859	0.05659	0.008606	0.075
41	PE(38:5)	0.220585	0.423242	0.480781	0.724608	0.071702	0.084021	0.193875	0.101452	0.210202	0.178325	0.102969	0.082757	0.1
42	[illegible]	[illegible]	[illegible]	[illegible]	[illegible]	[illegible]	[illegible]	[illegible]	[illegible]	[illegible]	[illegible]	[illegible]	[illegible]	[illegible]

Table S2. Peak area of each ceramide species identified in the plasma of healthy subjects (Control), TBE patients (TBE) and TBE patients co-infected with Lyme disease and Anaplasmosis (TBE+LD/HGA). Data obtained using MZmine software (XLSX).

	A	B	C	D	E	F	G	H	I	J	K	L	M	N	O	P	Q	R	S	T	U	V	W	X	Y	Z
1	Name	95	96	97	98	105	106	110	111	1	2	3	11	13	34	41	43	49	51	56	58	70	71	74	78	
2	Label	Control	Control	Control	Control	Control	Control	Control	Control	TBE	TBE	TBE	TBE	TBE	TBE	TBE	TBE	TBE	TBE	TBE	TBE	TBE	TBE	TBE	TBE	TBE-c
3	Cer(d16:2,	272546	321798.4	279013.7	263740.5	272696.7	226429.2	26496.96	245055.2	127976.3	89365.22	71039.25	95058.36	112493.2	125416.8	70152.45	28012.84	18963.98	149758.8	84884.46	165703.4	165800	97318.59	95939.77	109574	180
4	Cer(d16:2,	1484449	1257309	874848.6	1587436	1462978	31802.81	73867.47	207306.6	46144.28	2301.525	606063.8	13964.29	35094.34	45221.39	1877.18	103057.9	3329.17	382128	2271.388	41648.56	20607.68	176002.2	198779	12857.5	1801
5	Cer(d18:1,	234963.6	212609.5	216114.4	211536.7	212161.1	44657.98	181489.6	260106.9	5838.91	17606.69	252522.5	71021.75	73440.69	5722.132	3425.34	8660.755	2634.59	223252	4144.661	22726.63	239754.7	190364.6	291715	10657.31	2132
6	Cer(d18:2,	3157832	1858148	1747352	3903791	3884062	34718.77	136926.4	119983.7	64432.62	12423.57	3565278	49758.72	46048.76	63143.97	19192.69	106476.6	28244.52	3026050	23223.15	110116.9	173396.6	2095188	203735.9	48228.54	4272
7	Cer(d16:2,	261246.2	117003.1	150837.4	102670.2	75297.09	68698.22	145675.4	48364.89	141922.5	8730.61	71749.69	109700.4	79178.26	139084	63589.38	65079.78	37800.37	375820.4	76943.15	135451.7	523938.5	473194.3	435467.3	24594.11	788
8	Cer(d15:2,	187992.7	194795.2	183302	162332.2	159015.7	19583.39	273817.4	62802.42	4956.935	129176.2	291667.5	239421.1	201679.4	4857.796	120642.5	421414.7	29941.75	283595.3	145977.5	284908.2	7643.875	241883.1	472022.6	63530.76	2834
9	Cer(d18:1,	3199961	1764162	2028788	3649084	4542210	29346.94	203263.2	244618.6	1130.21	21028.12	4500297	38298.7	24916.57	1107.606	1928.675	91765.66	25359.73	3136279	2333.697	12171.26	383988.4	2916287	408010.6	4971.345	5157
10	Cer(d16:2,	400672.7	461557	448757	237393.6	251430	63160.91	291969.9	2246095	10090.97	84847.05	285751.3	27878.71	37398.78	9889.151	100996.1	129009	92109.89	281332.9	122205.3	77792.21	1034059	379880	392182.9	56440.71	3836
11	Cer(d18:1,	1812859	1468787	951609.5	2106489	1843966	108877.7	180180.8	296534.4	10585.23	1599.57	2550079	47895.83	48304.41	10373.53	6063.785	51968.13	6036.275	1577644	7337.18	887.425	324066.8	1241655	391819.9	10983.46	2687
12	Cer(d16:2,	338803	182771.4	164245.6	170083.7	169426.3	50003.68	80708.16	258687.5	11675.84	34381.21	203465.8	104431.8	222473.1	11442.32	19732.87	121248.8	9388.355	19984.77	23876.77	32610.45	636747.3	459664	438891.2	67519.77	3638
13	Cer(d18:2,	8664161	4924839	3514035	8815348	1.01E+07	224987.6	30867.91	43622.53	37623.23	13664.93	9078896	50792.64	31046.21	36870.77	5064.625	31971.45	3581.125	6537220	6128.196	81279.42	283467.8	6266934	45160.97	14466.61	1.04E
14	Cer(d16:1,	852055.5	630808.1	507649.5	731765.8	711070.5	36268.78	23177.84	4274.855	42830.23	21541.05	584879.1	16709.87	42039.62	41973.63	2503.045	58157.54	31067.43	446175.1	3028.684	100879.6	81011.43	265055.2	198790.8	2850.735	7230
15	Cer(d18:1,	506806.7	387919.1	323112.5	571254	503234.3	5367.97	2566.98	33145.05	3220.83	2554.88	558716.7	22123.24	1744.665	3156.413	3537.59	29562.1	4064.095	413132.4	4280.484	9900.52	170276.4	241812.2	30205.7	4702.365	7758
16	Cer(d18:1,	1450877	1078670	1089436	1092742	1015006	50307.69	25764.02	48997.12	10420.84	12825.26	776301.6	10247.17	38550.52	10212.42	4780.19	23742.95	1707.59	604221.7	5784.03	78233.87	92111.49	332792.7	333209.1	12655.86	8274
17	Cer(d18:1,	454901.5	389639.5	350261.4	417700.5	428771.6	43579.67	12373.04	6992.23	41811.08	8569.78	399949.4	52333.9	89158.22	40974.86	14012.31	104392.2	20968.22	281824.6	16954.9	52037.51	201308.5	143960.2	244313.6	4192.84	5268
18	Cer(d18:1,	193320.4	163230.4	154267.6	200390.6	187020.3	219756.2	188056.1	222780.3	65623.97	18021.06	31251.51	77626.73	120558.9	64311.49	99374.3	181164.9	118961.2	264155.3	120242.9	156660.2	86589.47	202907	186751.8	52846.45	2269
19	Cer(d18:2,	233283.4	242979	237849.4	127824.3	135794.5	42574.08	15826.19	12864.12	30887.9	9116.23	197486.7	35065.92	93210.68	30270.14	15447.97	30689.95	35375.44	171287	18692.04	39886.01	82468.05	17380.9	38240.67	13760.01	2323
20	Cer(d18:1,	137218.7	132860.4	213827.7	184001.7	170414.2	37440.24	197902.8	44861.36	7587.935	1314.99	356295.9	74913.98	68353.73	7436.176	3913.94	2923.545	2680.655	265043.5	4735.867	35172.9	190869.8	288706.6	170217.6	847.725	2471
21	Cer(d18:2,	221003.1	237464	235541	128573	140172.9	55512.09	43469.61	27259.61	69948.34	119151.8	239730.2	141748.7	161967.3	68549.37	43789.24	80061.48	12363.95	200745.9	52984.98	65727.24	227566.7	157295.8	23249.38	94155.28	1941
22	Cer(d18:1,	235628.4	250022.4	299159	199958.1	216251.5	92997.67	68435.4	31274.06	3044.175	60500.93	336311.7	110854.2	18688.47	2983.292	5415.95	58106.64	13049.99	309530	6553.3	92424.33	17957.69	286267.9	113867.3	67746.55	2067
23	Cer(d18:1,	243844.3	254681	251687.9	147998.8	160750.4	36851.4	16457.31	12031.97	1487.76	13314.68	276882.8	53060.44	84697.25	1458.005	10403.66	113762.7	3356.3	272536.3	12588.42	38730.42	139942.3	202847.9	127111.7	6066.21	2300
24	Cer(d18:2,	14171.86	68616.02	60265.59	48137.13	34275.71	118171.6	47724.94	33046.57	194696.3	30981.83	302710.2	81929.95	54107.02	190802.4	39723.54	163916.9	167926.8	342675.6	48065.48	155397.9	34218.96	285078	146190.1	51089.46	3443
25	Cer(d18:2,	348771.2	18007.04	372482.6	205217.6	4594.605	62371.31	40081.52	12505.37	89618.36	78757.01	313303	126543	111705	87825.99	73046.44	157562.4	10776.5	302273.8	88386.19	18230.25	33988.66	371192.3	181861	74135.82	2671
26	Cer(d16:1,	115064.8	654765.2	115793.3	422746.1	175973	59725.19	56261.93	404579.7	26440.16	15236.26	710640.3	66103.58	102518.9	25911.36	22697.28	105271.4	24476.97	600385.2	27463.7	28022.02	312941.8	837526.3	290823.3	8823.28	1217
27	Cer(d18:1,	56803.36	88034.19	55280.66	56045.72	40927.73	55306.98	56058.68	23598.47	10451.27	9874.045	273843.7	111523.7	136148.2	10242.24	5397.395	113190.8	31363.49	241496.5	6530.848	6914.47	306000.1	337117.2	25664.83	26812.94	3858
28	Cer(d16:2,	6208.68	19567.83	9945.695	57016.5	4920.73	9022.665	22489.93	3909.56	5944.18	36559.69	142159.2	13102.27	15262.43	5825.296	23920.32	102922.3	4233.44	104030.8	28943.58	58184.09	85364.5	194092.3	117390.2	28584.47	174
29	Cer(d16:2,	1.11E+07	5767963	6200820	1.56E+07	1.44E+07	88329.19	146914.4	48465.57	38984.19	1937.045	5830587	28436.09	57862.92	38204.5	15782.25	18004.44	76035.38	16146.22	19096.52	19457.5	302825.2	254070.9	272388.5	34853.72	1.42E
30	Cer(d18:0,	63026.44	39110.99	22998.66	70842.32	78326.95	30567.81	2772609	3350172	7250.955	38466.76	101881.1	274575.4	285710.8	8918.675	3379.58	2381.765	33451.52	139251.9	337727.8	6720.76	1506633	890240.7	2472200	21240.46	1702
31	Cer(d18:0,	94575.51	98782.16	25652.06	81689.87	75410.22	8543.615	570973.7	498062.7	69855.22	52906.32	284478.8	49047.13	28519.01	85921.92	3783.57	85104.76	40879.5	282773.9	60327.97	29375.77	585028.6	772758	539350.8	6094.205	106
32	Cer(d18:0,	47601.15	2558.435	37453.58	37035.4	20585.51	38911.84	425121.1	492011.5	60682.41	1230.23	164284.9	150084.5	205146.4	74639.36	2101.505	37276.49	55944.59	286614.9	184604	43060.57	464423.8	276781.6	579418.2	3137.465	7435
33	Cer(d18:0,	120620.9	47621.01	22908.27	107796.7	115138	8430.25	452276.3	435284.1	27229.31	6081.695	254086.6	12999.05	20652.39	33492.05	8529.26	59997.1	19329.33	131681.2	15988.83	24817.73	539932.3	201225.1	468805.4	26227.92	1854
34	Cer(d18:0,	1712788	169705.6	239889.6	1137849	694561.9	165306.9	364314.3	425431.1	70397.67	57969.4	1746674	24196.63	26255.56	86589.13	50866.44	160996.5	79867.93	1412562	29761.85	151853.5	1153455	487163.3	683328.3	34706.18	2065
35	Cer(d18:0,	9625.495	14861.27	22578.28	9609.285	6213.195	182588.2	61723.76	29899.09	10343.49	35232.38	31646.33	125109.7	136552.1	12722.49	1655.425	29336.26	5778.1	20112.09	153885	24125.37	175668	55541.93	51355.01	6388.5	1131
36	Cer(d18:0,	552406.8	435474.5	373286.7	688849.9	647229.3	5491.565	50033.71	74790.15	110526.4	28815.54	687475.4	34400.42	22687.76	135947.4	3204.065	130865.8	146201.6	481613.9	42312.51	131045	236120.3	654633.9	213150.1	44869.77	6374
37	Cer(d18:0,	571792.4	324129.9	340378.6	194765.8	165337	69072.66	60689.36	72107.21	24427.21	40751.09	520852.7	11109.48	32594.38	30045.46	7796.095	131911.1	81329.88	384848.4	13664.65	60827.64	238875.5	647160.8	188820.9	30460.26	2890
38	Cer(d18:0,	620507.1	194671	263547.3	340642.7	358542.9	37108.72	16719.9	10666.13	119055.9	105683.9	510238.9	76314.77	38865.23	146438.8	8233.99	81805.26	91471.85	248447.7	93867.17	87614.47	1109017	348455	76068.07	6890.75	4431
39	Cer(d18:0,	270306.6	238388.7	108882.3	238352.3	233473.8	84177.09	50095.03	15833.65	78012.59	79983.54	312555	6285.695	99656.11	95955.49	2102.91	69392.68	64555.93	272252	7731.405	107383.3	396546.6	301595.2	265655.8	46854.72	2058
40	Cer(d18:0,	375000.5	454350.1	334967.9	150950.3	186257.4	50015.15	47474.93	33943.85	23532.83	15672.47	202836.4	63542	90477.13	28945.37	85880.27	171812.5	116749	218212.2	78156.66	146569.2	501725.7	1011981	959009.1	20087.84	3090

Table S3. LC-MS parameters of most abundant phospholipid species identified in the plasma of healthy subjects (Control), TBE patients (TBE) and TBE patients co-infected with Lyme disease and Anaplasmosis (TBE+LD/HGA). (phosphatidylcholine (PC), lyso-phosphatidylcholine (LPC), phosphatidylethanolamine (PE), lyso-phosphatidylethanolamine (LPE), phosphatidylinositols (PI), phosphatidylserine (PS), and sphingomyelin (SM)).

Phospholipid class	m/z	Retention time	Phospholipid specie
**PC**	792.5728	19.47	PC(32:0)
	816.5751	19.78	PC(34:2)
	844.6049	19.40	PC(36:2)
	818.5891	19.78	PC(34:1)
	840.5727	19.98	PC(36:4)
	842.5874	19.43	PC(36:3)
	864.5767	18.75	PC(38:6)
	866.5882	18.74	PC(38:5)
	844.5502	19.25	PCp(38:8)
	892.6068	18.45	PC(40:6)
	820.6068	19.85	PC(34:0)
	868.5507	18.64	PC(38:4)
	838.5596	19.02	PC(36:5)
	950.6229	18.42	PCp(46:11)
	852.612	19.01	PCp(38:4)
	894.619	18.53	PC(40:5)
	922.5985	18.64	PC(42:5)
	790.5616	19.93	PC(32:1)
	898.5982	18.79	PCp(42:9)
	846.5688	19.27	PC(36:1)
	870.5681	18.72	PC(38:3)
**LPC**	554.3441	21.72	LPC(16:0)
	578.3445	21.77	LPC(18:2)
	580.3629	21.49	LPC(18:1)
	582.3763	21.52	LPC(18:0)
**PE**	750.542	7.28	PEo(38:5)/PEp(38:4)
	742.5372	7.54	PE(36:2)
	748.5274	7.22	PEo(38:6)/PEp(38:5)
	766.5402	7.28	PE(38:4)
	722.513	7.32	PEo(36:5)/PEp(36:4)
	746.5141	7.24	PEo(38:7)/PEp(38:6)
	726.5441	7.56	PEo(36:3)/PEp(36:2)

	724.5286	7.49	PEo(36:4)/PEp(36:3)
	762.5092	7.28	PE(38:6)
	790.538	7.29	PE(40:6)
	764.5227	7.34	PE(38:5)
	774.5421	7.24	PEo(40:7)/PEp(40:6)
	752.5594	7.47	PEo(38:4)/PEp(38:3)
	772.5284	7.19	PEo(40:8)/PEp(40:7)
	776.5569	7.25	PEo(40:6)/PEp(40:5)
	720.4987	7.64	PEo(36:6)/PEp(36:5)
	698.5118	7.87	PEo(34:3)/PEp(34:2)
**LPE**	476.2765	10.10	LPE(18:2)
	478.2925	10.00	LPE(18:1)
	500.2766	9.63	LPE(20:4)
	452.2792	10.05	LPE(16:0)
	480.3083	9.97	LPE(18:0)
	524.2782	9.71	LPE(22:6)
**PI**	885.5477	4.61	PI(38:4)
	861.5511	4.65	PI(36:2)
	887.5651	4.54	PI(38:3)
	857.5171	4.73	PI(36:4)
	863.5642	4.72	PI(36:1)
	835.5349	4.76	PI(34:1)
**PS**	816.5751	16.78	PS(38:1)
	818.5891	16.78	PS(38:0)
	840.575	15.98	PS(40:3)
	844.6049	16.40	PS(40:1)
	868.6061	15.67	PS(42:3)
	842.5921	16.24	PS(40:2)
	864.5767	15.75	PS(42:5)
	866.5882	15.74	PS(42:4)
	846.6243	16.39	PS(40:0)
	870.6232	15.87	PS(42:2)
	892.6068	15.45	PS(44:5)
	838.5596	16.02	PS(40:4)
	894.619	15.53	PS(44:4)
	790.5616	16.93	PS(36:0)
**SM**	761.5809	19.37	SM(d34:1)
	871.6912	18.36	SM(d42:2)
	845.675	18.49	SM(d40:1)
	843.6606	18.58	SM(d40:2)
	869.6725	18.28	SM(d42:3)

	789.6127	19.23	SM(d36:1)

Table S4. LC-MS parameters of most abundant ceramide species (CER[NS], CER[NDS]) identified in the plasma of healthy subjects (Control), TBE patients (TBE) and TBE patients co-infected with Lyme disease and Anaplasmosis (TBE+LD/HGA). *(non-hydroxy fatty acid [N], dihydrosphingosine [DS] and sphingosine [S]).*

Ceramide class	m/z	Retention time	Ceramide specie
CER[NS]	678.6656	43.17	Cer(d18:1/26:0)
	620.589	37.85	Cer(d18:2/22:0)
	550.5141	42.34	Cer(d15:2/20:0)
	648.6224	41.70	Cer(d18:1/24:1)
	650.6377	41.68	Cer(d18:1/24:0)
	622.608	36.79	Cer(d18:1/22:0)
	594.575	39.95	Cer(d18:1/20:0)
	536.4954	42.81	Cer(d18:2/16:0)
	676.652	43.48	Cer(d18:1/26:1)
	562.5127	43.93	Cer(d18:2/18:1)
	564.5269	41.84	Cer(d18:1/18:1)
	538.5112	44.13	Cer(d18:1/16:0)
	590.5441	41.35	Cer(d18:2/20:1)
	592.5573	44.47	Cer(d18:2/20:0)
	566.5417	44.27	Cer(d18:1/18:0)
	564.5318	35.01	Cer(d16:2/20:0)
CER[NDS]	650.6379	33.26	Cer(d18:0/24:1)
	596.5908	31.68	Cer(d18:0/20:0)
	652.6532	33.38	Cer(d18:0/24:0)
	624.623	34.20	Cer(d18:0/22:0)
	678.662	24.77	Cer(d18:0/26:1)
	540.524	29.30	Cer(d18:0/16:0)
	568.5581	33.74	Cer(d18:0/18:0)
	566.5417	33.72	Cer(d18:0/18:1)

Table S5. KEGG pathway analysis of lipid metabolic pathways to TBE-Control and TBE+LD/HGA-Control

Batch	Pathway Name	Match Status	p	-log(p)	Holm p	FDR
TBE vs Contol	Sphingolipid metabolism	1/21	3.2811E-10	9.484	1.9686E-9	1.9686E-9
	Glycerophospholipid metabolism	4/36	1.5096E-9	8.8211	7.5482E-9	4.5289E-9
	Glycosylphosphatidylinositol (GPI)-anchor biosynthesis	1/14	0.0026419	2.5781	0.010568	0.0052838
	Arachidonic acid metabolism	1/36	0.020941	1.679	0.062822	0.020941
	Linoleic acid metabolism	1/5	0.020941	1.679	0.062822	0.020941
	alpha-Linolenic acid metabolism	1/13	0.020941	1.679	0.062822	0.020941
TBE+LD/HGA vs Contol	Glycerophospholipid metabolism	4/36	1.0279E-8	7.9881	6.1673E-8	6.1673E-8
	Arachidonic acid metabolism	1/36	7.3742E-8	7.1323	3.6871E-7	1.1061E-7
	Linoleic acid metabolism	1/5	7.3742E-8	7.1323	3.6871E-7	1.1061E-7
	alpha-Linolenic acid metabolism	1/13	7.3742E-8	7.1323	3.6871E-7	1.1061E-7
	Sphingolipid metabolism	1/21	0.08599	1.0656	0.17198	0.10319
	Glycosylphosphatidylinositol (GPI)-anchor biosynthesis	1/14	0.64783	0.18854	0.64783	0.64783

Examples of MSMS spectra of some phospholipid species

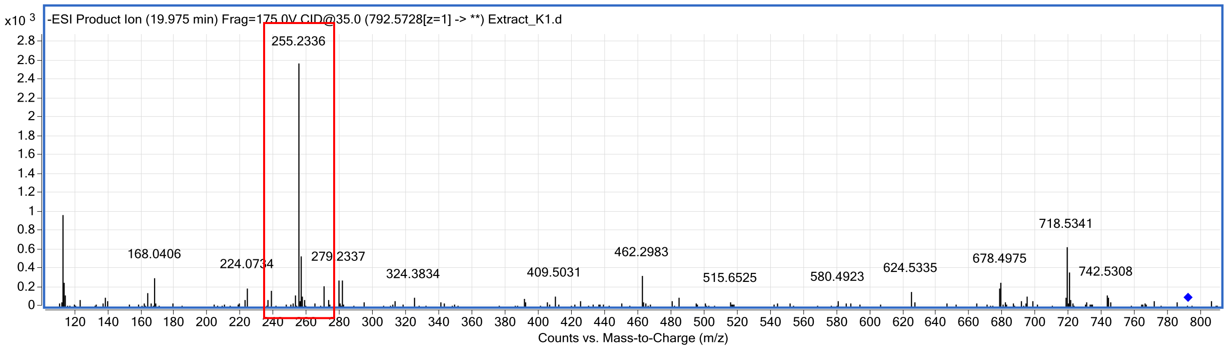

Figure S1. ESI-MS/MS spectrum of the [M+CH_3COO]$^-$ ion of **PC(16:0/16:0)** [**m/z 792.5728**]. **Characteristic fragment ions** are: **m/z 255.2336** corresponding to [M-H]$^-$ ion of palmitic acid (16:0).

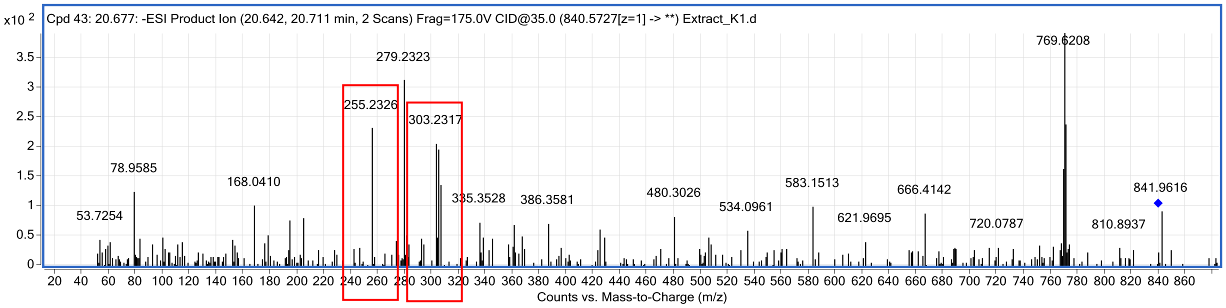

Figure S2. ESI-MS/MS spectrum of the [M+CH_3COO]$^-$ ion of **PC(16:0/20:4)** [**m/z 840.5727**]. **Characteristic fragment ions** are: **m/z 255.2326** corresponding to [M-H]$^-$ ion of palmitic acid (16:0) and **m/z 303.2317** corresponding to [M-H]$^-$ ion of arachidonic acid (20:4).

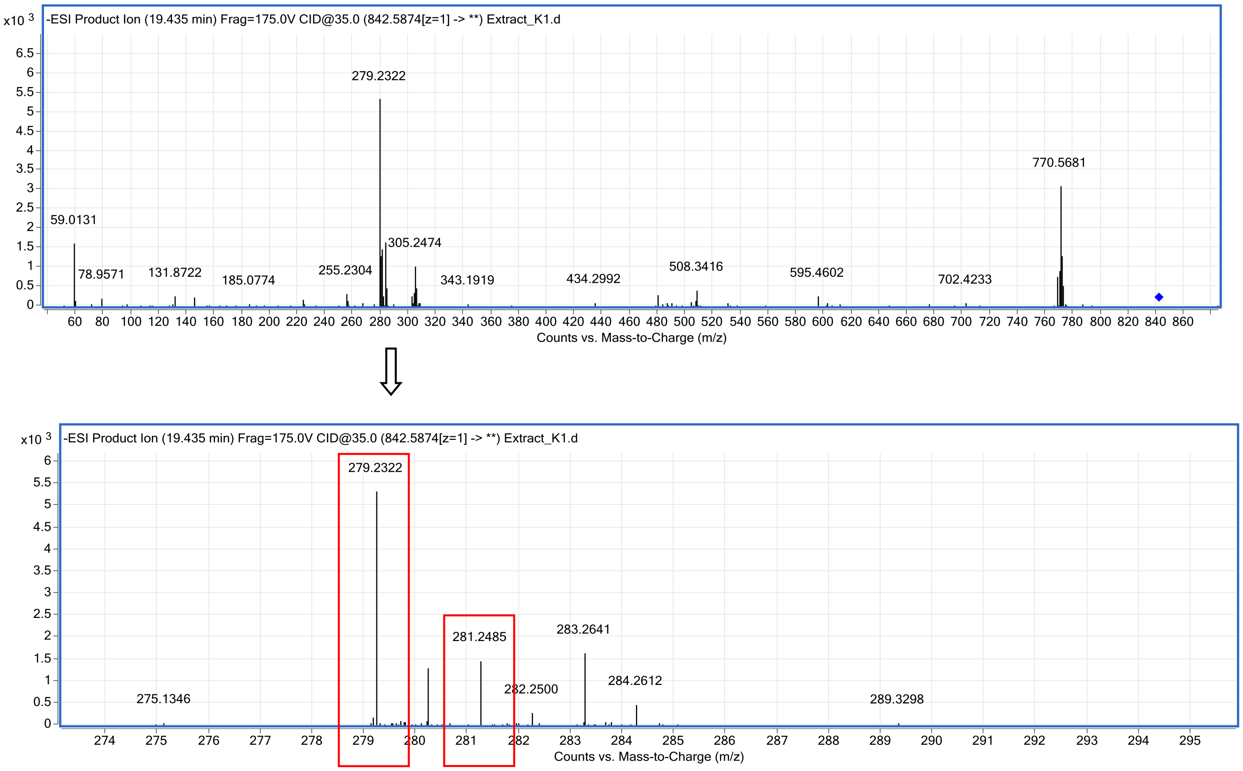

Figure S3. ESI-MS/MS spectrum of the [M+CH_3COO]$^-$ ion of **PC(18:1/18:2)** [**m/z 842.5874**]. **Characteristic fragment ions** are: **m/z 279.2322** corresponding to [M-H]$^-$ ion of linoleic acid (18:2) and **m/z 281.2485** corresponding to [M-H]$^-$ ion of oleic acid (18:1).

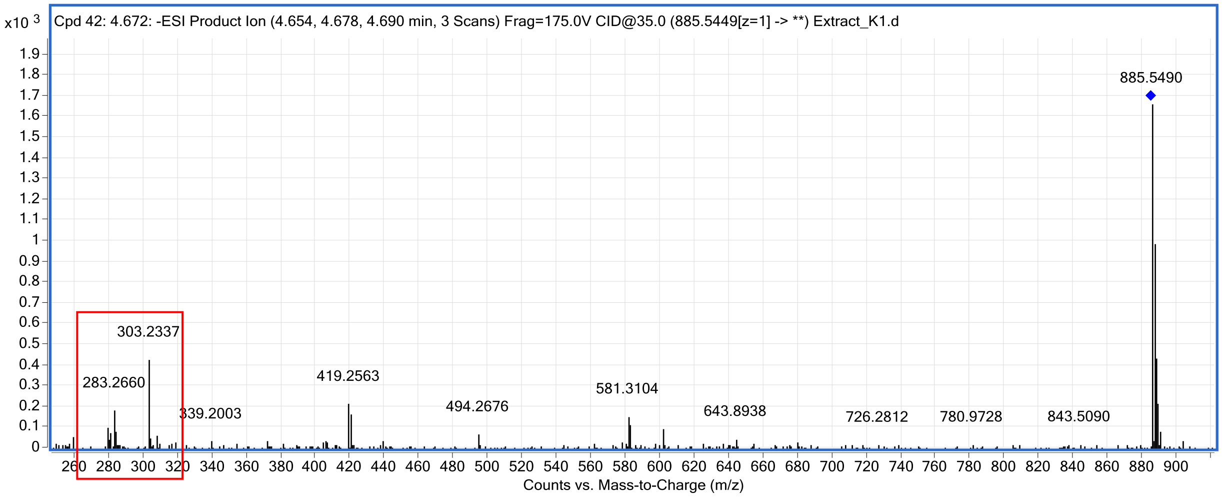

Figure S4. ESI-MS/MS spectrum of the $[M-H]^-$ ion of **PI(18:0/20:4)** [**m/z 885.5490**]. **Characteristic fragment ions** are: **m/z 283.2660** corresponding to $[M-H]^-$ ion of stearic acid (18:0) and **m/z 303.2337** corresponding to $[M-H]^-$ ion of arachidonic acid (20:4).
